# Deregulated microRNAs in neurofibromatosis type 1 derived malignant peripheral nerve sheath tumors

**DOI:** 10.1038/s41598-020-59789-4

**Published:** 2020-02-19

**Authors:** Azadeh Amirnasr, Robert M. Verdijk, Patricia F. van Kuijk, Pinar Kartal, Anne L. M. Vriends, Pim J. French, Martin E. van Royen, Walter Taal, Stefan Sleijfer, Erik A. C. Wiemer

**Affiliations:** 1 Department of Medical Oncology, Erasmus MC Cancer Institute, University Medical Center Rotterdam, Erasmus MC, Rotterdam, The Netherlands; 2 Department of Pathology, University Medical Center Rotterdam, Erasmus MC, Rotterdam, The Netherlands; 3 Department of Neurology, Cancer Treatment Screening Facility (CTSF), University Medical Center Rotterdam, Erasmus MC, Rotterdam, The Netherlands; 4 Department of Pathology, Cancer Treatment Screening Facility (CTSF), Erasmus Optical Imaging Centre (OIC), University Medical Center Rotterdam, Erasmus MC, Rotterdam, The Netherlands; 5 Department of Neuro-Oncology/Neurology, University Medical Center Rotterdam, Erasmus MC, Rotterdam, The Netherlands

**Keywords:** Sarcoma, Cell invasion, Sarcoma

## Abstract

Malignant peripheral nerve sheath tumors (MPNST) are aggressive cancers that occur spontaneously (sporadic MPNST) or from benign plexiform neurofibromas in neurofibromatosis type 1 (NF1) patients. MPNSTs metastasize easily, are therapy resistant and are frequently fatal. The molecular changes underlying the malignant transformation in the NF1 setting are incompletely understood. Here we investigate the involvement of microRNAs in this process. MicroRNA expression profiles were determined from a series of archival, paired samples of plexiform neurofibroma and MPNST. Ninety differentially expressed microRNAs were identified between the paired samples. Three downregulated microRNAs (let-7b-5p, miR-143-3p, miR-145-5p) and two upregulated microRNAs (miR135b-5p and miR-889-3p) in MPNST were selected for functional characterization. In general, their differential expression was validated in a relevant cell line panel but only partly in a series of unpaired, fresh frozen tumor samples. As part of the validation process we also analyzed microRNA expression profiles of sporadic MPNSTs observing that microRNA expression discriminates NF1-associated and sporadic MPNSTs. The role of microRNAs in cancer progression was examined in NF1-derived MPNST cell lines by transiently modulating microRNA levels. Our findings indicate that some microRNAs affect migratory and invasive capabilities and Wnt signaling activity but the effects are distinct in different cell lines. We conclude that miRNAs play essential regulatory roles in MPNST facilitating tumor progression.

## Introduction

Neurofibromatosis type 1 (NF1) is a relatively common autosomal dominant disorder which is caused by inherited or sporadic mutations in the *NF1* gene^1–3^. The *NF1* gene encodes the tumor suppressor neurofibromin 1 that functions as a negative regulator of Ras signaling by its GTPase- activating protein (GAP) domain. The partial inactivation of neurofibromin 1 seen in NF1 patients can cause variable symptoms affecting the skin, bone and the nervous system. Moreover, the disease is associated with an increased risk of benign and malignant tumor formation. Almost all NF1 patients develop cutaneous neurofibromas and in many instances also deeper seated plexiform neurofibromas. These benign tumors are believed to originate from the Schwann cell lineage i.e. mature Schwann cells or Schwann cell precursors^4,5^ and are characterized by a biallelic inactivation of the *NF1* gene^1,6^. Approximately 10% of NF1 patients develop malignant peripheral nerve sheath tumors (MPNSTs) usually in the context of pre-existing plexiform neurofibromas. MPNST are highly aggressive tumors that are largely responsible for the reduced life expectancy these patients face^7–9^. Early metastasis, poor prognosis, and resistance to therapeutic interventions are common clinical features of this cancer. While patients with non-metastatic disease may benefit from surgical resection and radiation, many patients relapse. These patients, and also those initially presenting with advanced disease, face a poor prognosis as there are only a limited number of systemic agents available for these patients such as doxorubicin, ifosfamide and pazopanib. The relatively modest anti-tumor activity of these agents translates in a median overall survival of approximately one year^10,11^. A better understanding of the essential molecular mechanisms underlying plexiform neurofibroma transformation to MPNST is crucial to reveal NF1 patients who are at risk to develop MPNST and to identify new targets for treatment.

MicroRNAs (miRNAs) are a class of small non-protein coding RNAs of approximately 19–26 nucleotides in length that function in post-transcriptional gene regulation. They generally operate by binding, in the context of the RNA induced silencing complex (RISC), to the 3′ untranslated region of target mRNAs. MiRNA binding, through base pairing between the miRNA and mRNA, cause mRNA degradation and/or inhibition of translation^12,13^. Over the past two decades it became clear that miRNAs fulfil pivotal roles in a wide variety of biochemical and physiological processes and are intimately involved in numerous pathological processes including cancer^14–17^. A dysregulated miRNA expression profile is a key characteristic of cancer and can be exploited for diagnostic purposes. There is ample evidence that miRNAs can have oncogenic or tumor suppressive properties. However, in many instances the extent to which individual – aberrantly expressed – miRNAs contribute to carcinogenesis and cancer progression and/or affect treatment response is not fully understood. A limited number of miRNA profiling studies examined human neurofibroma and NF1-derived MPNST tumor samples and implicated the involvement of several miRNAs in the malignant transformation of plexiform neurofibroma to MPNST^18–21^. Although of interest, these studies are difficult to compare as different miRNA detection platforms were used, variable numbers of unpaired tumor samples were examined and only a few miRNAs were functionally characterized. Here we analyzed miRNA expression, using an established and highly reproducible RT-PCR procedure, in a unique series of paired human archival tumor samples of plexiform neurofibroma and MPNST. Each individual neurofibroma/MPNST pair being derived from the same NF1 patient. The expression of a selected set of differentially expressed miRNAs was validated using a well-characterized neurofibroma/MPNST cell line panel as well as fresh frozen samples of plexiform neurofibromas, atypical neurofibromas and MPNST. To understand how these miRNAs affect carcinogenesis and/or MPNST progression we modulated their expression levels in MPNST cell lines and assessed their impact on cellular proliferation, migration and invasion and Wnt/β-catenin signaling.

## Materials and Methods

### Tumor samples

Neurofibromatosis type 1 patients from which both archival plexiform neurofibroma as well as MPNST resection samples were available were identified in the Erasmus Medical Center patient files. Formalin-fixed paraffin-embedded (FFPE) tissue blocks were collected, from the Erasmus MC Tissue bank, of a set of nine neurofibroma-MPNST pairs (see Supplementary Table 1 for patient and tumor characteristics). In addition, ten FFPE tumor tissue blocks were collected that were derived from patients diagnosed with sporadic MPNST (Supplementary Table 2). Fresh frozen tumor samples from plexiform neurofibroma (n = 7), atypical neurofibroma (n = 4) and NF1-associated MPNST (n = 11) (Supplementary Table 3) from the Erasmus MC tissue bank were included for validation purposes. Hematoxylin-eosin-stained sections of these samples were examined by an expert pathologist at the Erasmus MC (RMV) to confirm the initial histopathological diagnosis using criteria as described before^22,23^ in accordance with the 2016 WHO classification of Tumours of the Central Nervous System^24^. The experimental protocol was submitted for review to, and approved by, the Medical Ethics Committee Erasmus MC of Rotterdam (MEC-2016-213). All experimental procedures, including the use of human tissues samples, were performed in accordance with the relevant guidelines and regulations, with all researchers adhering to the code of conduct for medical research as laid out by the council of the Federation of Dutch Medical Scientific Societies (https://www.federa.org/codes-conduct). The use of anonymous or coded left-over material for scientific purposes is part of the standard treatment agreement with patients and therefore informed consent was not required according to Dutch law.

### Cell culture

The human NF1-associated MPNST derived cell lines 90-8TL, ST88-14 and the sporadic MPNST derived STS26T cell line were a kind gift of Dr. Eduard Serra (Institute of Predictive and Personalized Medicine of Cancer/IMPPC, Barcelona, Spain). The sNF96.2 cell line (NF1-derived MPNST) and the HS53T cell line (cutaneous neurofibroma) were obtained from the American Type Culture Collection (ATCC). All cell lines were routinely cultured in Dulbecco’s modified Eagle’s medium (DMEM) (Gibco Life Technologies) supplemented with 10% fetal bovine serum (FBS), 100 IU/ml penicillin and 100 µg/ml streptomycin at 37 °C in a humidified atmosphere containing 5% CO_2_. All cell cultures were regularly screened for mycoplasma infection. Short Tandem Repeat (STR) profiles of the cell lines were established for authentication purposes (Supplementary Fig. 1) and were matched to source profiles at the ATCC, Deutsche Sammlung von Mikroorganismen und Zellkulturen (DSMZ) or the literature when available.

### RNA isolation

Total RNA was extracted from 5–6 20 µm sections from each FFPE tumor sample using the RecoverAll^TM^ total nucleic acid isolation kit (Ambion/Life Technologies) according to the manufacturer’s recommendations. RNAbee (Tel test Inc.) was used to isolate total RNA from cell pellets and fresh frozen tumor tissue samples following standard protocols. The quality and concentration of all the RNA preparations were examined using a Nanodrop-1000 (Nanodrop Technologies).

### MicroRNA profiling

The miRNA expression profiles were determined in FFPE samples using TaqMan® Low Density Array (TLDA) Human MicroRNA Cards (A card v2.0, B card v3.0; Applied Biosystems/Thermo Fisher Scientific) capable of detecting 756 distinct human miRNAs essentially as previously described^25^. In brief: Two pools of cDNA were prepared using Megaplex™ RT Primers Human Pools (pool A v2.1, pool B v3.0) and a Taqman® microRNA reverse Transcription kit (Applied Biosystems/Thermo Fisher Scientific). Next, a pre-amplification step was carried out using Megaplex™ PreAmp Primers Human Pools (pool Av2.1, pool B v3.0) together with the Taqman™ PreAmp master-mix (Applied Biosystems/Thermo Fisher Scientific). The resulting products were further amplified using Taqman™ Universal PCR Master-Mix No AmpErase® on human microRNA A and B cards in a 7900HT Fast Real-Time PCR system (Applied Biosystems/Thermo Fisher Scientific). The expression (C_T_ value) of a specific miRNA in a sample was normalized to the median C_T_ of all detectable miRNAs in that sample. Subsequently the normalized relative expression (2^−ΔCT^) was calculated for each miRNA. The normalized miRNA expression data were log 2 transformed and median centered to acquire the relative expression values that were used for hierarchical clustering analyses using Cluster-3.0 and Java TreeView for visualization. The clustering was based on the uncentered correlation as a distance metric using average linkage. A Student T-test (paired) was used to determine statistical significance between distinct groups of expression data and the Benjamini-Hochberg false discovery rate (FDR) was used to control for multiple testing.

### RT-PCR

The expression level of individual miRNAs was determined using the TaqMan^®^ MiRNA Assays Technology (Applied Biosystems/Thermo Fisher Scientific) in a neurofibroma/MPNST cell line panel and fresh frozen tumor samples. In brief: Total RNA (50 ng) was reverse transcribed in a multiplex reaction using specific miRNA primers from the TaqMan^®^ MiRNA Assays and reagents from the TaqMan^®^ MiRNA Reverse Transcription Kit (Applied Biosystems/Thermo Fisher Scientific) according to the manufacturer’s protocol. The resulting cDNA was used as input in a quantitative real-time PCR (qPCR) using a miRNA specific primer/probe mix together with the TaqMan^®^ Universal PCR Master Mix No AmpErase^®^ UNG (Applied Biosystems/Thermo Fisher Scientific) using the 7500 Fast Real-Time PCR systems (Applied Biosystems/Thermo Fisher Scientific). The qPCR data were analyzed using SDS software (version 2.4, Applied Biosystems/Thermo Fisher Scientific). A standard dilution series of a cDNA sample-pool was included on every plate allowing for the absolute quantification of the miRNA expression.

The mRNA expression of Wnt target genes was determined by RT-PCR using the TaqMan^®^ Technology (Applied Biosystems/Thermo Fisher Scientific). In brief: Total RNA (1 µg) was used as input for a reverse transcription reaction using a high capacity cDNA reverse transcription kit (Applied Biosystems/Thermo Fisher Scientific) according to protocols of the manufacturer. The cDNA was used as input in a PCR reaction using primer/probe combinations from the following Taqman® gene expression assays (LEF1, assay ID: Hs01547250_m1; MSX2, assay ID: Hs00741177_m1; SOX9, assay ID:Hs00165814_m1; TWIST1, assay ID: Hs00361186_m1) and Taqman® Universal PCR master mix using the 7500 Fast Real-Time PCR system (all obtained from Applied Biosystems/Thermo Fisher Scientific) according to the manufacturer’s recommendations. Three housekeepers (GAPDH, HPRT and PPIA) were used for normalization purposes using the comparative C_T_-method^26^. The qPCR data were analyzed using SDS software (version 2.4, Applied Biosystems/Thermo Fisher Scientific).

### Transfections

Human MPNST cells were plated in 96-well plates at a concentration of 2–18 × 10^3^ cells/well (SNF96.2); 2–10 × 10^3^ cells/well (ST88–14) and 2–10 × 10^3^ cells/well (90-8TL) in a total volume of 200 μl of standard cell culture medium without antibiotics. After 24 h cells were transfected with 50 nM MiRIDIAN microRNA mimics (Dharmacon) of let7b-5p, miR-143-3p, miR-145-5p and miR-29c-3p or 50 nM MiRCURY LNA^TM^ inhibitors (Exiqon) of miR-135b-5p and miR-889-3p. As controls a scrambled miRNA mimic Negative control #1 (Dharmacon) and the miRCURY LNA^TM^ inhibitor Negative Control (Exiqon) were used. DharmaFECT I was used as a transfection reagent. Transfection conditions were optimized (transfection efficiency > 90%) for each of the cell lines using a fluorescently labelled miRNA mimic (miRIDIAN mimic transfection control Dy547; Dharmacon) (Supplementary Fig. 2).

### Proliferation assay

Twenty-four hours prior to transfection, cells were plated in a 96-well plate. The next day the cells were transfected at approximately 40–50% confluency with selected miRNA mimics/inhibitors or appropriate controls. Cell viability was assessed by a sulforhodamine B (SRB) assay at 72 h post-transfection essentially as described previously^27^. In short: cells were fixed by 10% TCA in PBS, washed and stained by 0.4% SRB in 1% acetic acid for 15 min, washed in 1% acetic acid and dried. Color was dissolved in Tris-Base after which the A_540nm_ was measured using a spectrophotometer.

### Migration assays

#### Wound healing kinetics

Twenty-four hours prior to transfection, cells were plated in a 96-well ImageLock^TM^ plate (Essen BioScience Ltd.). The next day the cells were transfected with selected miRNA mimics/inhibitors or appropriate controls. At 24 h post-transfection, all 96 wells were scratched simultaneously in the central axis of the individual wells using the WoundMaker^TM^ (Essen Bioscience Ltd.). A live-cell imaging system, IncuCyte (Essen BioScience Ltd.) was used to automatically monitor the kinetics of cell migration every 2 hours for a total duration of 26 h during which cells migrate from the scratch edges into the wound area.

#### Cell-speed measurements

Twenty-four hours prior to transfection, cells were plated in a 96-well CellCarrier^TM^-96 Ultra microplate (PerkinElmer). The next day the cells were transfected with selected miRNA mimics or appropriate controls. At 24 h post-transfection cells were imaged at 2 h intervals in an Opera Phenix™ HCS system (PerkinElmer) for 40 h. Software (Harmony® High Content Imaging and Analysis Software, PerkinElmer) was used to calculate the average cell speed of the individual cells in the wells of the microplate.

### Invasion assay

Cells were cultured in a 6-well plate and transfected with selected miRNA mimics/inhibitors or appropriate controls. At 24 h post-transfection the cells were harvested by mild trypsinization, resuspended in DMEM supplemented with 2.5% FBS and plated into the IncuCyte^TM^ ClearView 96-well insert (Essen BioScience Ltd.) at a concentration of 2 × 10^3^ cells/well (SNF96.2); 2 × 10^3^ cells/well (ST88–14) and 7.5 × 10^3^ cells/well (90-8TL). Prior to plating the transfected cells, the IncuCyte ClearView insert membranes were coated with 50 μg/ml Matrigel (BD Biosciences). The inserts were subsequently placed in a 96-well plate containing DMEM supplemented with 10% FBS and incubated under standard cell culture conditions. An IncuCyte live-cell imaging system (Essen BioScience Ltd.) was used to capture cell invasion monitoring and quantifying invading cells through the matrigel coated membranes every two hours for a total period of 67 h.

### Wnt reporter assay

Wnt/β-catenin signaling activity was determined by a β-catenin/TCF reporter assay in a two-step transfection process. In brief: SNF96.2, ST88-14 and 90-8TL cell lines were plated in 24-well plates in culture medium without antibiotics. When the cells reached 60–70% confluency they were transfected with MiRCURY LNA^TM^ inhibitors (Exiqon) of miR-135b-5p, miR-889-3p or a miRCURY LNA^TM^ inhibitor Negative Control (Exiqon) in a final concentration of 50 nM using Dharmafect I. After 24 h the cells were co-transfected with 250 ng of the TOP-Flash or FOP-Flash firefly luciferase reporter constructs^28^ and 25 ng of a SV40-Renilla luciferase expression (for normalization purposes) using FuGene ®HD (Promega). Eight hours post-transfection the cells were stimulated with 25% L-control medium in DMEM or 25% L-Wnt3A medium in DMEM and left to incubate for 16 h after which the cells were lysed and assayed for firefly and Renilla luciferase activities using the Dual-Luciferase reporter assay system (Promega).

## Results

### Plexiform neurofibromas can be distinguished from MPNST by their microRNA expression profile

**Figure 1:**
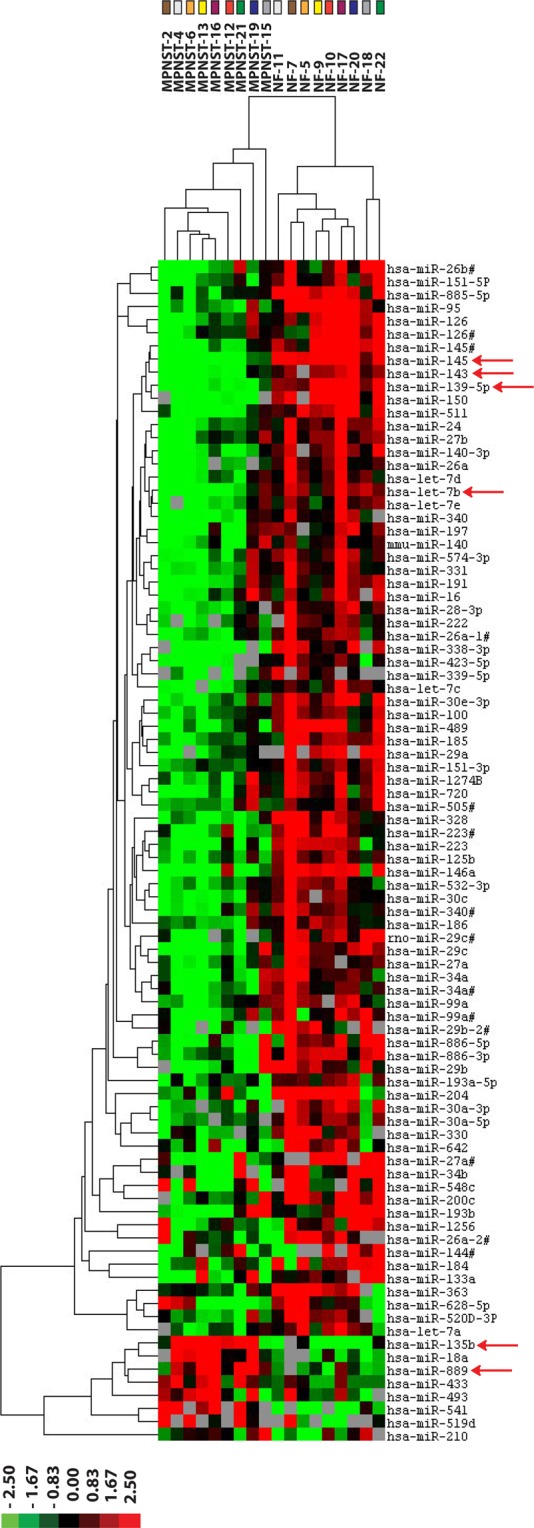
Differentially expressed microRNAs between plexiform neurofibromas and MPNST. The miRNA expression profiles were determined of FFPE sections from nine paired sets of plexiform neurofibroma (NF) and MPNST tumor samples, each pair derived from the same NF1 patient that initially presented with a plexiform neurofibroma and at a later stage developed an MPNST. Depicted are the results of a supervised hierarchical clustering using the most differentially expressed miRNAs (p < 0.025, FDR < 10%) in the analyses. The color code on top indicates the relative position of the neurofibroma-MPNST pairs in the cluster tree. The heat map lists the individual miRNAs and their relative expression levels in the MPNST and neurofibroma clusters. Red arrows indicate miRNAs that were selected for further validation and functional studies. In the heat map red indicates relative high expression, green relative low expression, grey designates missing expression values.

To study the involvement of miRNAs in the malignant transition of benign plexiform neurofibromas into MPNST we determined the miRNA expression profiles of a unique series of nine paired plexiform neurofibromas and MPNST samples. Each plexiform neurofibroma/MPNST pair was derived from the same NF1 patient. An unsupervised hierarchical clustering, based on the expression of all detectable miRNAs in these paired samples, already grouped most of the benign plexiform neurofibromas and MPNSTs in distinct clusters (Supplementary Fig. 3). A supervised clustering analysis using the 90 most significant differentially expressed miRNAs (p < 0.025; FDR < 10%) between neurofibromas and MPNSTs grouped the samples into clearly separate clusters (Fig. 1, Supplementary Table 4). The majority (82 out of 90; 91%) of the differentially expressed miRNAs were found to be downregulated in the MPNST group in comparison to the plexiform neurofibromas. The downregulated miRNAs include members of well-known cancer related miRNA clusters like the miR-23/27/24 clusters on chromosome 9-q22.32 and 19-p13.12, the miR-143/145 cluster on 5-q33.1, the miR-29b-1/29a and miR-29b-2/29c clusters on chromosome 7-q32.3 and 1-q32.2, respectively. In addition, 5 members of the let-7 family, let-7a/b/c/d/e were found downregulated in MPNST. Only 8 (9%) of the miRNAs exhibited a higher expression in the MPNST samples than in neurofibromas, these include miR-135b, miR-889, miR-493, miR-433 and miR-541, the last four all belonging to a large miRNA cluster on the long arm of chromosome 14 (14-q32.31). Particularly, miR-135b and miR-541 are aberrantly expressed in the MPNST setting with a 52-fold and 20-fold upregulation, respectively.

### Validation of differentially expressed microRNAs between plexiform neurofibromas and MPNST in a cell line panel and fresh frozen tumor samples

**Figure 2:**
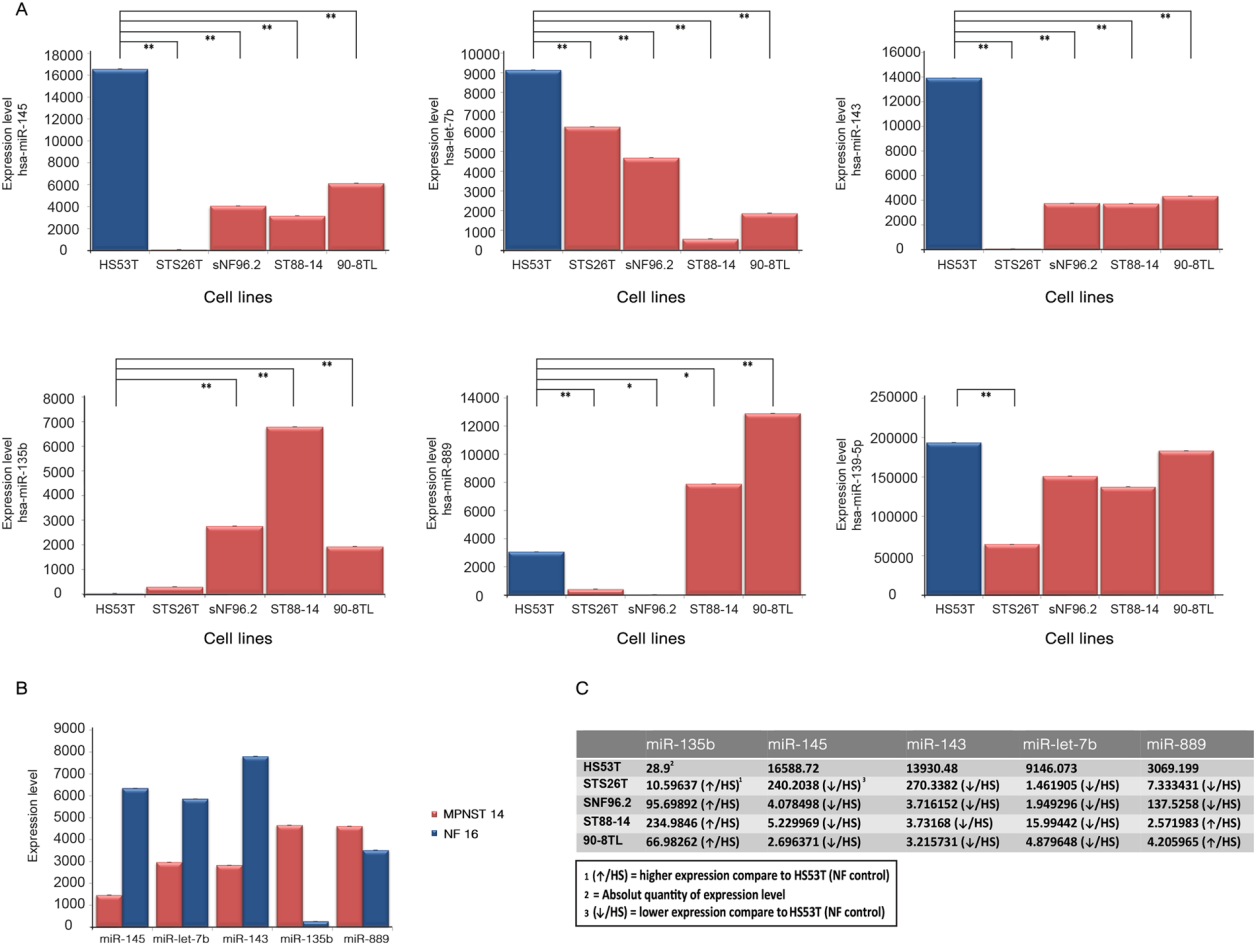
Expression of selected microRNAs in a neurofibroma/MPNST cell line panel and in a fresh frozen neurofibroma/MPNST sample pair. (**A**) Expression levels measured by quantitative RT-PCR of selected miRNAs (miR-145-5p, let-7b-5p, miR-143-3p, miR-135b-5p, miR-889-3p and miR-139-5p) in a well-characterized cell line panel consisting of a cutaneous neurofibroma cell line (HS53T), NF1-associated MPNST cell lines (SNF96.2, ST88-14, and 90-8TL) and a sporadic MPNST cell line (STS26T). Bars depict average values ± SD (n = 3–4). A T-test was used to determine statistical significance; *p-value < 0.05, **p-value < 0.01. (**B**) Expression level of selected miRNA in a fresh frozen plexiform neurofibroma/MPNST sample pair derived from the same NF1 patient. (**C**) Summary of the expression level fold-changes of selected miRNAs in the MPNST cell lines compared to the expression level observed in the cutaneous neurofibroma cell line HS53.T.

**Figure 3:**
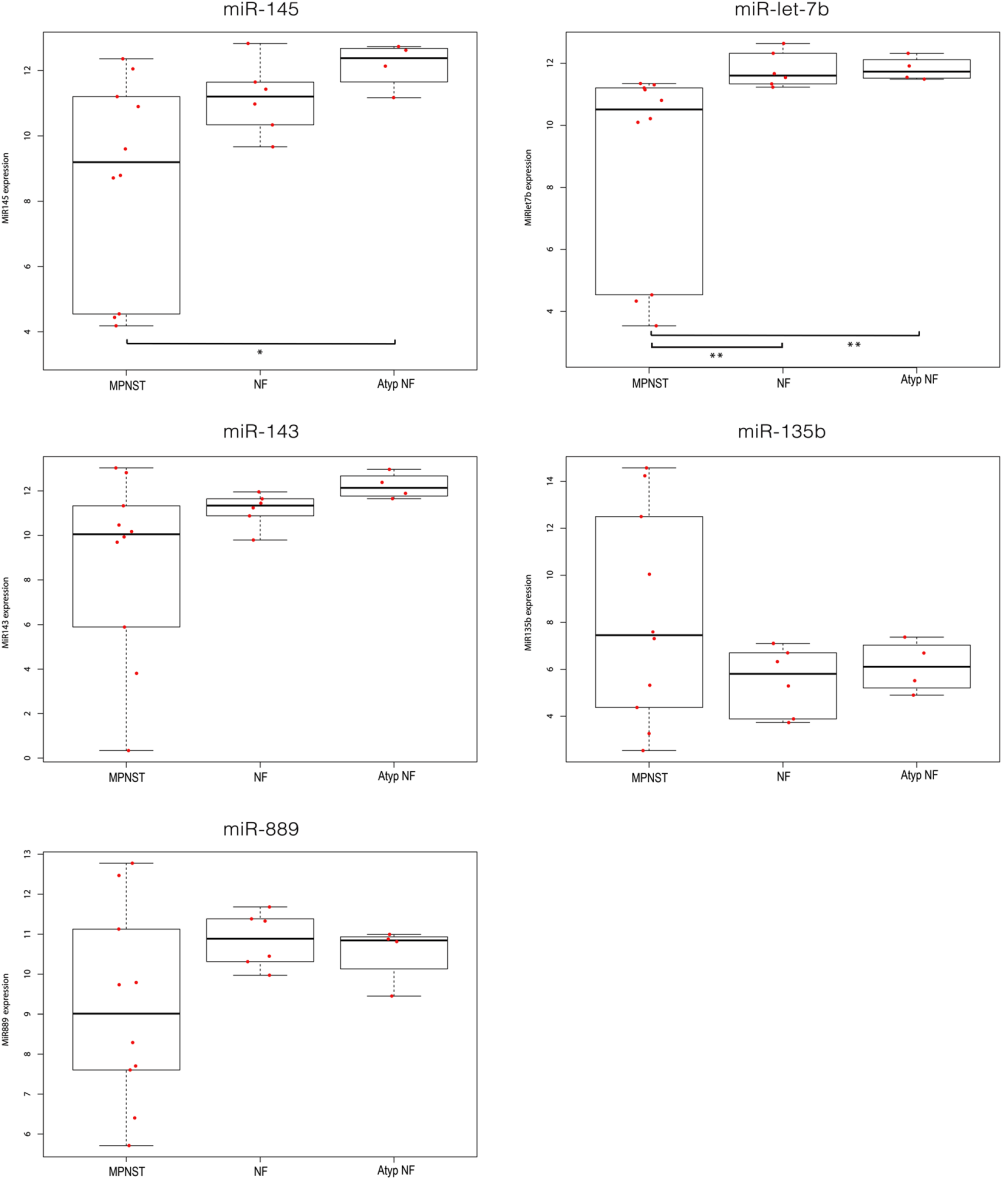
Expression level of selected microRNAs in unpaired fresh frozen plexiform neurofibroma, atypical neurofibroma and MPNST samples. A quantitative RT-PCR was used to determine miRNA levels of miR-145-5p, let-7b-5p, miR-143-3p, miR135b-5p and miR-889-3p in unpaired fresh frozen NF1-derived MPNST (n = 10), plexiform neurofibroma (NF; n = 6) and atypical neurofibroma (Atyp NF; n = 4). Relative expression is depicted using Box-Whisker plots with boxes showing 1^st^ to 3^rd^ quartile with the median marked by a horizontal line. A Mann Whitney U test was used to determine statistical significance; **p-value < 0.01, *p-value < 0.05.

It was investigated whether the differential expression of a selected set of miRNAs could be validated in a well-characterized cell line panel and additional, unpaired fresh frozen neurofibroma and MPNST samples. Taking statistical significance (p < 5 × 10^−4^; FDR < 1%), fold-difference (>3 in at least 75% of the sample pairs) into account as well as the reported involvement of miRNAs in cancer, we selected the following miRNAs for further validation and subsequent functional studies miR-145-5p, miR-143-3p, miR-139-5p and let-7b-5p all downregulated in MPNST and miR-135b-5p and miR-889-3p as representatives of the upregulated miRNAs (Fig. 1, Supplementary Tables 4 and 5). When considering the expression of the selected miRNAs in the individual NF-MPNST samples pairs it was noted that the fold-difference of the up or down-regulation varies considerably between different pairs (Supplementary Table 5). Using quantitative PCR, we could validate the expression of the selected miRNAs as shown in Fig. 2A. MiR-145, let-7b, miR-143 - and to a lesser extent miR-139-5p - were all downregulated in the MPNST cell lines STS26T, sNF96.2, ST88-14 and 90-8TL compared to their expression level in a cutaneous neurofibroma cell line Hs53.T. Conversely, miR-135b was found upregulated in MPNST cell lines. MiR-889 was clearly upregulated in ST-88-14 and 90-8TL but downregulated in sNF96.2 and the sporadic MPNST cell line STS26T. In general, these results (Fig. 2C) confirm our miRNA profiling findings and identify the MPNST cell lines as representative models for this malignancy. As the expression distribution between neurofibroma and MPNST for miR-139-5p reflected our profiling results the least, we omitted this miRNA from further analyses. As additional validation we determined the expression levels of the selected miRNAs in fresh frozen samples from a plexiform neurofibroma/MPNST sample pair derived from the same patient (Fig. 2B). In agreement with our previous observations we demonstrated downregulation of miR-145, miR-143 and let-7b whereas miR-135b and miR-889 were upregulated in the MPNST sample. We also determined the expression levels of the selected miRNAs in a larger unpaired panel of fresh frozen tumor samples consisting of plexiform neurofibromas (n = 6), atypical neurofibromas (n = 4) and MPNSTs (n = 10) (Fig. 3). The expression level of the miRNAs in atypical neurofibroma samples is not significantly different from the expression observed in plexiform neurofibromas. A comparison between the miRNA expression levels in MPNST and neurofibromas indicated a significant downregulation in the MPNST group of let-7b (p < 0.01) and of miR-145 when the expression levels in MPNST were compared to levels in atypical neurofibromas (p < 0.05). A comparison of miR-145 levels between MPNST and plexiform neurofibromas was borderline significant (p = 0.0572). Likewise, the expression of miR-143 between MPNST and atypical neurofibromas was borderline significant (p = 0.0584). No significant statistical difference between sample groups was observed for miR-889, miR-143 and miR-135b expression. The high variability observed in the expression levels of the selected miRNAs, particularly in the MPNST samples, most likely reflects tumor heterogeneity and may obscure differences. This problem may be partly overcome by analyzing paired samples.

### NF1-associated MPNST and sporadic MPNST display distinct miRNA expression profiles

**Figure 4:**
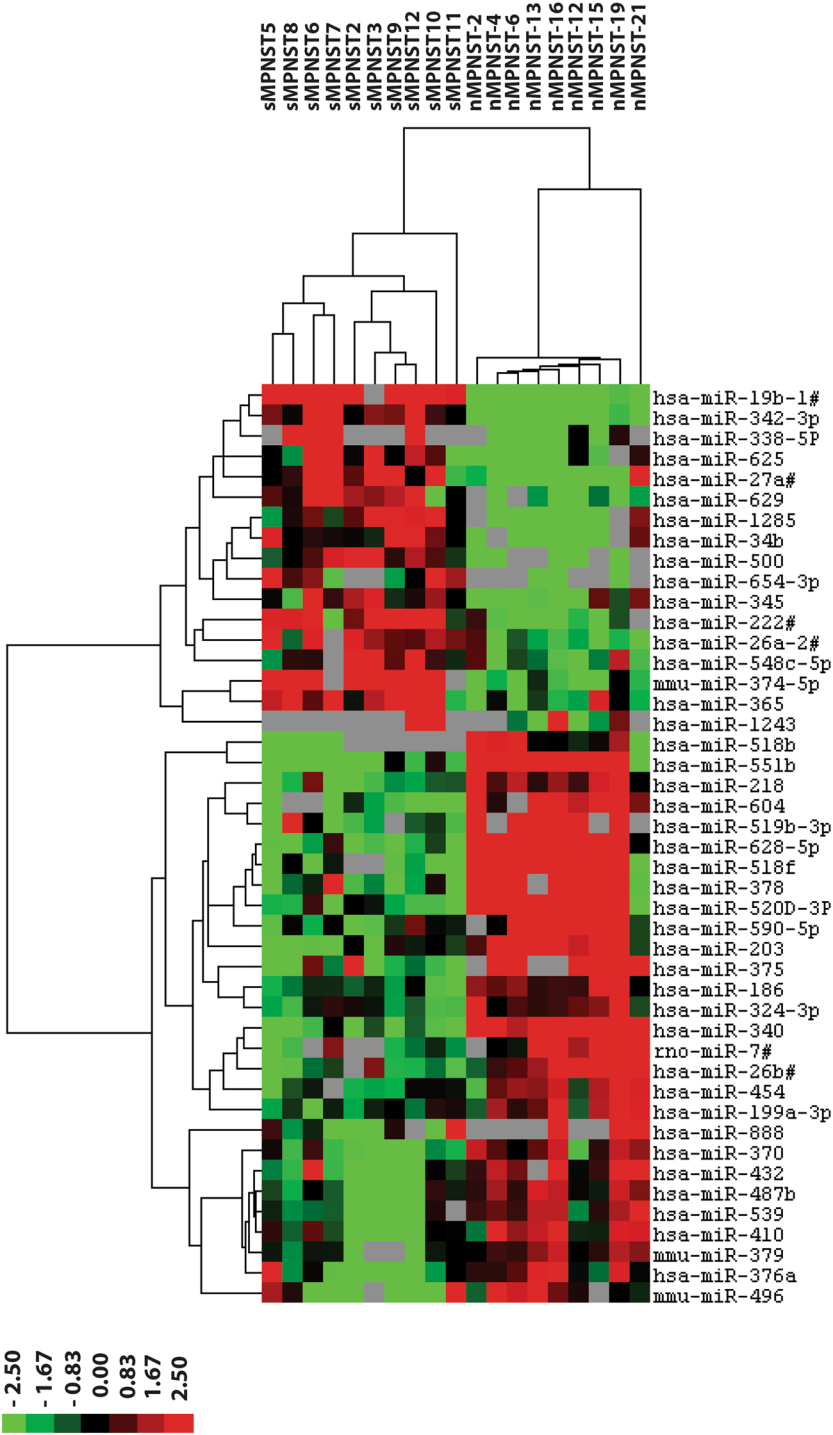
Differentially expressed microRNAs between NF1-derived and sporadic MPNST. The miRNA expression profiles derived from FFPE sections of sporadic MPNST (sMPNST) (n = 10) are compared with the miRNA profiles from NF1-derived MPNST (n = 9, see Fig. 1). Depicted is a supervised hierarchical clustering using the most significant differentially expressed miRNAs (p < 0.011, FDR < 10%). The heat map lists the miRNAs and their relative expression levels. The differences indicate the different etiology of sporadic and NF1 derived MPNST and may reflect a different biology. In the heat map red indicates relative high expression, green relative low expression, grey designates missing expression values.

To assess whether the selected miRNAs are specifically dysregulated in NF1-derived MPNST, we examined miRNA expression in 10 archival sporadic MPNST samples. A comparison between the miRNA profiles observed in the sporadic MPNST and the NF1-derived MPNST revealed many differentially expressed miRNAs (Fig. 4, Supplementary Table 6, Supplementary Fig. 4) emphasizing these tumor types have a different etiology and possibly a different biology. A cluster analysis using the 45 most significantly differentially expressed miRNAs (p < 0.03, FDR < 10%) completely discriminated the two MPNST types (Fig. 4). Very few of the miRNAs identified in the plexiform neurofibroma-MPNST comparison, and none of the selected miRNAs, were detected in the sporadic MPNST – NF1-derived MPNST comparison. Apparently, the selected miRNAs were not differentially expressed between sporadic and NF1-derived MPNST. Therefore, we cannot rule out that the selected miRNAs are also aberrantly expressed in sporadic MPNST and play a role in carcinogenic processes in these tumors as well.

### MicroRNAs affect migratory and invasive capacity of MPNST cell lines

The selected miRNAs that are dysregulated in NF1-associated MPNST may contribute to the process of tumorigenesis and metastasis. All have been linked to various aspects of carcinogenesis in other cancers. The clustered miR-143 and miR-145 genes are widely regarded as tumor suppressors in epithelial tumors^29–32^ and were indicated as having a critical role in tumor stroma^33,34^. MiR-135b has been implicated in the progression of several cancers^35–37^ and let-7b is considered a tumor suppressor miRNA^38^. *In vitro* experiments were conducted to examine the functional role of the selected miRNAs and their effect on cellular proliferation, migration and invasion. To that end, transiently, the expression levels of let-7b, miR-145 and miR-143 were restored and miR-135b and miR-889 levels were reduced, in MPNST cell lines. As a control we included a miR-29c mimic. This miRNA was reported by Presneau *et al*. to be reduced in MPNST and to affect migration and invasion, but not proliferation^20^.

**Figure 5:**
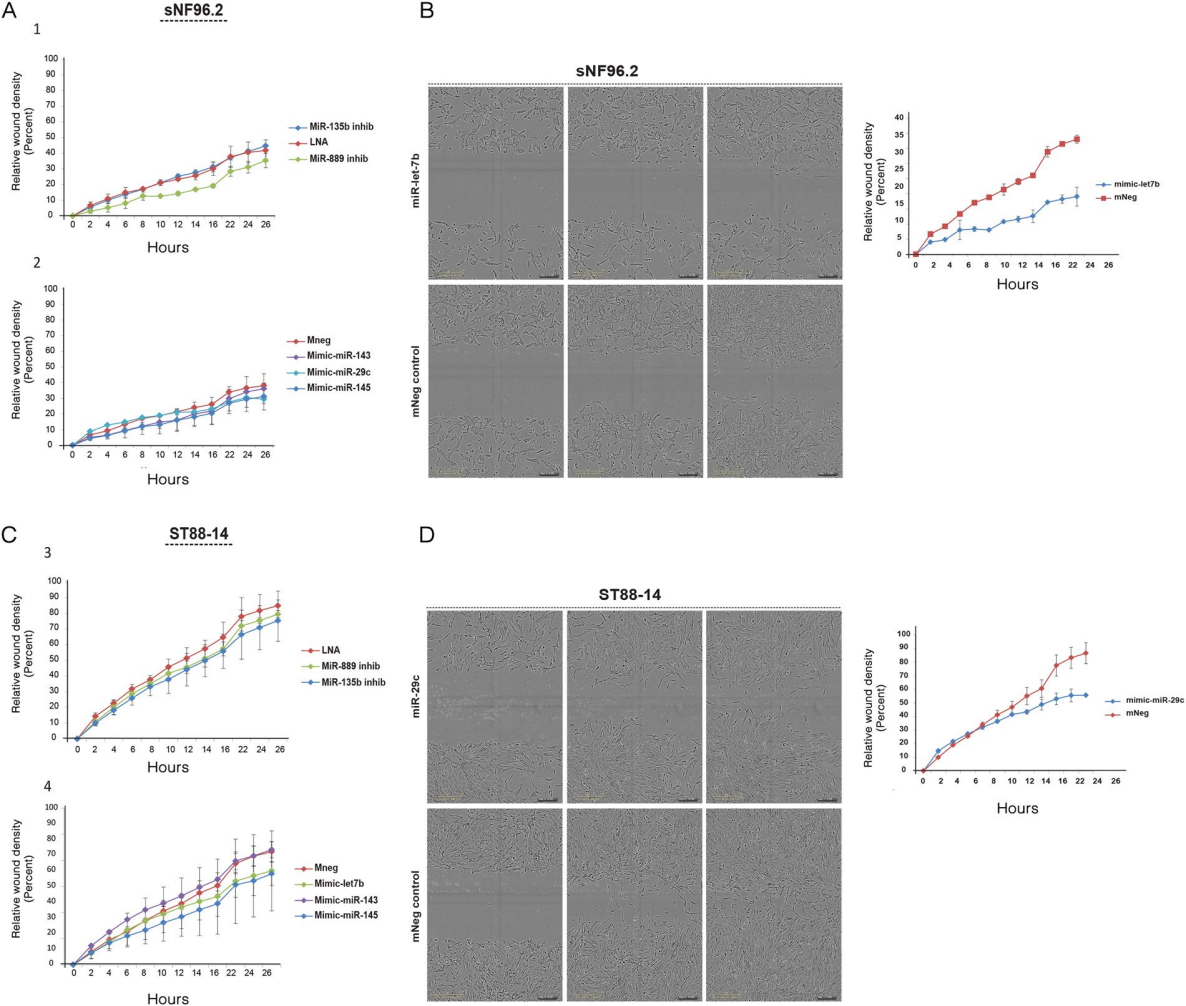
Effects of selected microRNAs on cell migration of the MPNST cell lines sNF96.2 and ST88-14. The NF1-associated MPNST cell lines sNF96.2 and ST88-14 were transfected with scrambled (LNA control), miR-135b and miR-889 inhibitors or with a scrambled (mneg control), miR-143, miR-145, let-7b and miR-29c mimics. (**A**,**C**) Scratch assay after which cell migration is monitored every two hours for 26 h using a live-cell imaging system (IncuCyte; Essen Bioscience Ltd.). (**B**,**D**) Micrographs illustrating the effects of let-7b and miR-29c mimics on cell migration in sNF96.2 and ST88-14, respectively. The individual panels show the situation directly after scratching (left panels), at 14 h (middle panels) and after 26 h (right panels). Depicted are representative images and graphs, in the graphs individual data points indicate average values ± SD (n = 3).

First, we focused on cellular proliferation using an SRB assay to assess the effects of miRNA modulation. By quantifying the amount of cells in time, comparing control transfections with miRNA mimic/inhibitor transfections we determined whether cell proliferation was affected. It was observed that none of the miRNA mimics or inhibitors significantly and consistently affected proliferation with the exception of miR-29c overexpression in sNF96.2 which stimulated cell proliferation (Supplementary Fig. 5). We next assessed whether the selected miRNAs affect the migratory and invasive capacity of the tumor cells. We performed a scratch assay to measure the migration potential of the transfected MPNST cells. Figure 5 depicts representative results on the kinetics of migration in sNF96.2 and ST88-14 transfectants obtained by a live-cell imaging system. Most miRNA mimics and inhibitors did not significantly interfere with the migratory capacity of the MPNST cells (Fig. 5A,C). However, a clear reduction of the migration rate was observed in sNF96.2 cells transfected with let-7b (Fig. 5B) and in ST88-14 cells transfected with miR-29c (Fig. 5D). None of the miRNA mimics and inhibitors had an effect on the migration capacity of 90-8TL cells (Supplementary Fig. 6A). These observations were confirmed when we determined the average cell speed as a measure for migratory capacity using a different cell imaging system (Supplementary Fig. 7).

**Figure 6:**
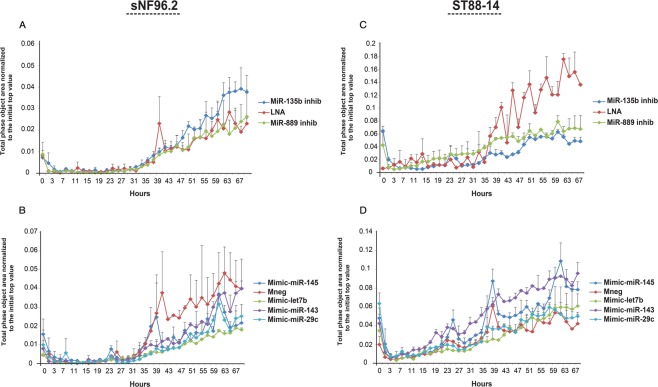
Effects of selected microRNAs on the invasive capacity of the MPNST cell lines sNF96.2 and ST88-14. (**A**–**D**) The NF1-associated MPNST cell lines sNF96.2 and ST88-14 were transfected with scrambled (LNA control), miR-135b and miR-889 inhibitors or with a scrambled (mneg control), miR-143, miR-145, let-7b and miR-29c mimics. Invasive capacity was monitored every two hours for 67 h using a live-cell imaging system (IncuCyte). Y-axis indicates the “Total phase object area normalized to the initial top value” as a measure for the invading cell population. Depicted are representative graphs individual data points indicate average values ± SD (n = 3). A Mann Whitney U test was used to determine statistical significance comparing the last 10 datapoints in each series.

Next, we examined the effect of the selected miRNAs on the invasive capacity of MPNST cells in a cell invasion assay. The MPNST cell lines sNF96.2 and ST88-14 were transfected and seeded onto 96-well invasion plates containing a matrigel coated membrane. Invasion of cells was quantitatively monitored by live-cell imaging in time. A strikingly reduced invasive capacity was observed in ST88-14 transfected with miR-135b (p < 0.01) and miR-889 (p < 0.03) inhibitors (Fig. 6C). These effects, however, were not seen in sNF96.2 transfectants (Fig. 6A) or 90-8TL transfectants (Supplementary Fig. 6B). In sNF96.2 cells, transfection with let-7b (p < 0.01) and miR-29c (p < 0.01) mimics, and to a lesser extent with miR-145 (p < 0.05) resulted in reduced invasiveness (Fig. 6B). In contrast, miR-143 (p < 0.01) and miR-145(p < 0.01) mimics appeared to boost invasion in ST88-14 (Fig. 6D). We conclude that miRNA modulation effects are cell line dependent.

### miR-135b and miR-889 modulate Wnt/β-catenin signaling in MPNST cells

**Figure 7:**
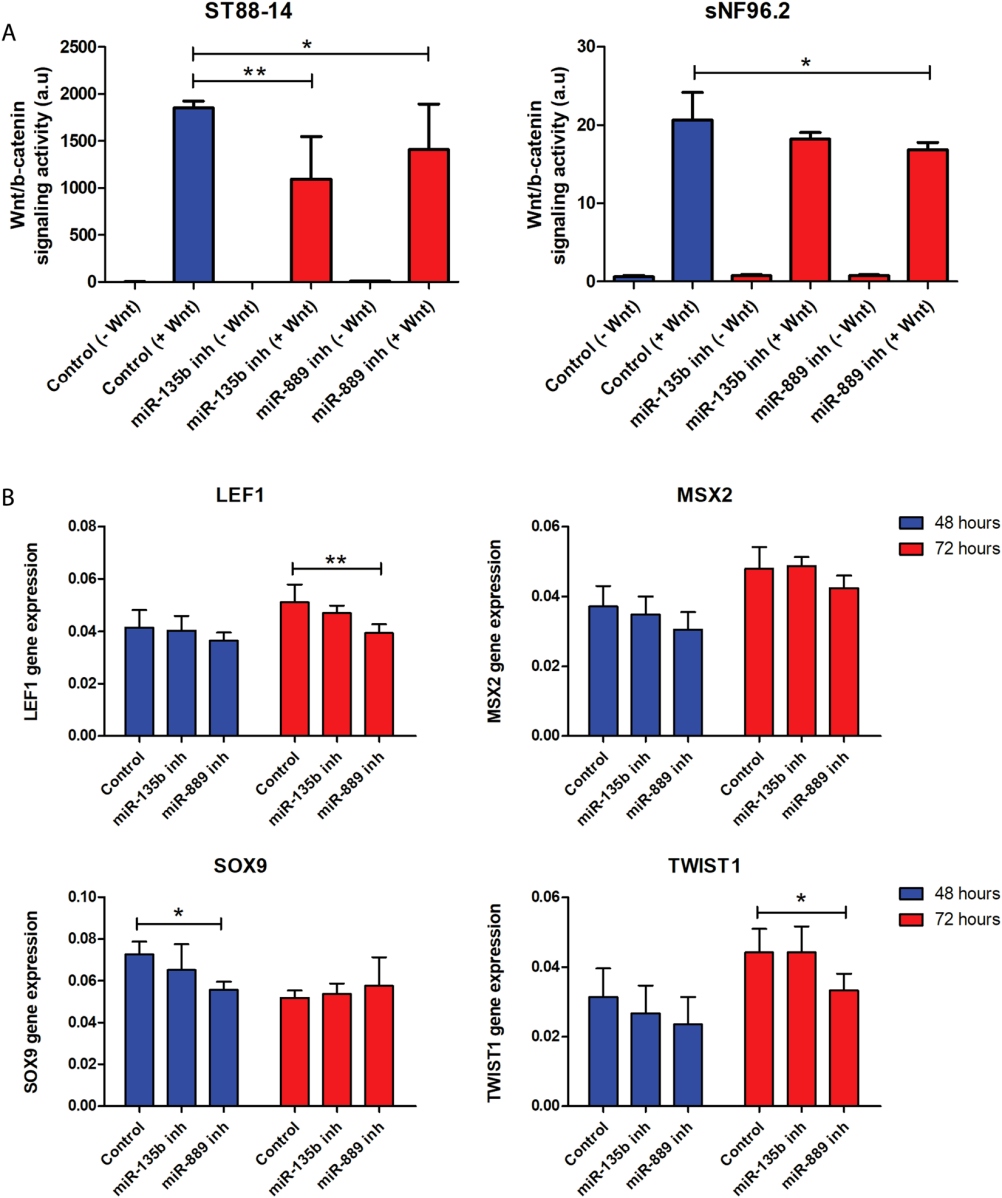
Effects of miR-135b and miR-889 inhibition on Wnt signaling capacity and Wnt target expression in MPNST cell lines. The NF1-associated MPNST cell lines ST88-14 and sNF96.2 were transfected with scrambled (LNA control), miR-135b and miR-889 inhibitors. (**A**) Wnt/ß-catenin signaling activity upon induction by Wnt ligand was determined using a β-catenin/TCF reporter assay. Depicted are average values ± SD (n = 9) (**B**) mRNA expression levels of Wnt target genes (LEF1, MSX2, SOX9, TWIST1) in ST88-14 cell line. Transcript levels were measured by quantitative RT-PCR at 48 h and 72 h after transfection with scrambled (LNA control), miR-135b and miR-889 inhibitors. Depicted are normalised average expression values ± SD (n = 4-6). In both (**A**,**B**) a Mann Whitney U test was used to determine statistical significance, *p-value < 0.05, **p-value < 0.01.

Recently the canonical Wnt/β-catenin signaling pathway has been identified as driver pathway of both benign neurofibromas and MPNST^39,40^. Moreover, miR-135b was reported to target multiple negative regulators of Wnt like Adenomatous Polyposis Coli (APC)^36^. Likewise, miR-889, another overexpressed miRNA in MPNST, was also predicted to target *APC* according to TargetScan version 7.1. We therefore examined whether miR-135b (on average 52x higher in MPNST) and miR-889 (on average 3x higher in MPNST) (Supplementary Tables 4 and 5) are capable of modulating Wnt signaling activity in the MPNST setting. sNF96.2, ST88-14 and 90-8TL were transfected with miR-135b or miR-889 inhibitors. Next, we determined Wnt/ß-catenin signaling activity using a TCF/ß-catenin reporter assay. It was noticed that Wnt/ß-catenin activity upon induction with Wnt ligand was highest in ST88-14 (Fig. 7A) and 90-8TL (Supplementary Fig. 6C) with relatively low Wnt activity being measured in sNF96.2 (Fig. 7A). Transient reduction of miR-135b and miR-889 expression significantly impaired the induction of Wnt/ß-catenin signaling activity in ST88-14 (Fig. 7A). No significant effects were observed in the 90-8TL despite the relatively high Wnt activity levels observed in this cell line (Supplementary Fig. 6C). A small but significant reduction of Wnt activation was seen in miR-889 inhibitor transfectants of sNF96.2 but not in the miR-135b inhibitor transfectant (Fig. 7A). To verify that Wnt signaling is indeed affected the mRNA expression levels of Wnt target genes LEF1, MSX2, SOX9 and TWIST1, all genes expressed in MPNST and Schwann cells^39^, were determined by quantitative PCR  (Fig. 7B). ST88-14 cells that display active Wnt signaling (Fig. 7A) were transfected with control, miR-135b and miR-889 inhibitors. Figure 7B indicates that miR-135b inhibition consistently showed a trend of lowering the expression level of the Wnt target genes compared to a control transfection although without reaching statistical significance. Inhibition of miR-889 gave rise to similar results but the reduction in expression of the Wnt targets seemed stronger, more persistant and reached statistical significance. We conclude that both the overexpressed miR-135b and miR-889 in NF1-associated MPNST may augment Wnt signaling activity.

## Discussion

MPNSTs are highly aggressive tumors with a dismal prognosis for those confronted with advanced disease^10,11^. Half of these tumors arise in the context of NF1 from benign pre-existing plexiform neurofibromas^7,41^. Genetic aberrations associated with this transformation include mutations in CDKN2A^42^ and TP53^43^ and the recently disovered loss-of-function mutations in SUZ12 and EED, essential components of the PRC2 complex^44–46^. However, the precise molecular mechanisms underlying this maligant transition are still unclear. We investigated the involvement of miRNAs in the tumorigenesis and progression of MPNST.

MiRNAs are intricately connected to cancer and play critical roles in cancer gene regulation and diverse aspects of tumorigenesis^14–17^. Until now only few studies addressed the miRNA involvement in neurofibroma and MPNST biology^18–21^. All studies reported clear differences in miRNA expression between plexiform neurofibromas and MPNST when unpaired tumor samples were analyzed. However, the functional significance and pathological roles of dysregulated miRNAs in the context of MPNST are not, or poorly studied. In 2010, Chai *et al*. were the first to point out the downregulation of let7a/b in MPNST cells and their effect on MPNST cell invasiveness^18^. We as well observed the downregulation of multiple let-7 family members, including let7a/b in MPNST and noticed that let-7b expression interfered with cellular migration and invasion in NF1-derived MPNST cell lines. Let-7 family members are known to target Ras^47^ it might therefore be that their relatively low levels in MPNST facilitate Ras signaling. Presneau *et al*. described a reduction of miR-29 members, most notably miR-29c, in MPNST^20^. They demonstrated that miR-29c played a role in tumor progression by controlling migration and invasion via the regulation of the matrix metalloproteinase-2 (MMP2)^20^. Our findings also indicated significantly lowered levels of miR-29a/b/c and we confirmed – although not in all NF1-derived MPNST cell lines – the effects of miR-29c on migration and invasion^48^. Supplementary Table 7 presents an overview of all miRNAs that were found dysregulated in NF1-associated MPNST in comparison to plexiform neurofibromas in multiple studies. Note that in general only few miRNAs were found upregulated in MPNST and that most differentially expressed miRNAs display a reduced expression level in MPNST.

By comparing the miRNA expression profiles of a unique series of paired samples of neurofibromas and MPNST, we defined a group of miRNAs that are aberrantly expressed in NF1-derived MPNST. From the 90 miRNAs that were identified we chose six miRNAs to examine their functional role in the pathogenesis of MPNST. MiR-143/145, let7b, miR-139-5p, miR135b, and miR-889 were among the top 15 of differentially expressed miRNAs (Supplementary Table 4). All, with the exception of miR-889, were also reported as misexpressed in MPNST by other researchers (Supplementary Table 7). However, none of the miRNAs we examined, with the exception of let-7b, has been studied in MPNST. We were able to validate the reduced expression of let-7b, miR-143/145 and the increased expression of miR-135b in MPNST using a relevant cell line panel. The upregulation of miR-889 was only observed in two NF1 associated MPNST cell lines (ST88-14 and 90-8TL) and not in sNF96.2 and the sporadic MPNST cell line STS26T. The increased expression of miR-889 in MPNST, which is less striking than that of miR-135b, may be more variable and occur only in a subset of tumors. When we examined the expression of the selected miRNAs in an unrelated series of unpaired fresh frozen plexiform neurofibroma, atypical neurofibroma and NF1-derived MPNST samples the down or upregulation of most selected miRNAs could not be firmly established. This may be due to the limited number of samples, together with the highly variable expression of the miRNAs examined in the MPNSTs. These results, however, do emphasize the value of paired samples and the need to analyze well-characterized and adequately sized cohorts to account for tumor heterogeneity.

A direct comparison between sporadic MPNST and NF1-derived MPNST revealed that these two tumor types could be completely distinguished on the basis of their miRNA expression profiles. This result contrasts with the findings of Holtkamp *et al*. who reported that sporadic and NF1-derived MPNST could not be distinguished by their mRNA expression patterns^49^. However, this study only examined the expression of 558 genes comparing 6 sporadic MPNSTs with 4 NF1-derived MPNST, due to its limited set-up differences may have been missed.

Functional experiments initially focused on proliferation, migration and invasion, all key elements of carcinogenesis and cancer progression. It was uncovered that the selected miRNAs did not affect proliferation but their overexpression (miR-143, miR-145 and let-7b) or inhibition (miR-135b, miR-889) interfered with migration and invasion although not all cell lines responded in a similar fashion and/or with equal intensity (Figs. 5 and 6; Supplementary Figs. 6 and 7). Recently Watson *et al*. implicated the Wnt/β-catenin signaling pathway to fulfil an essential role in both neurofibromas and MPNST showing that inhibition of Wnt signaling by small molecules reduced viability and induced apoptosis^40^. The precise biological basis of Wnt/β-catenin signaling activation is only partly known and involves the downregulation of members of the β-catenin destruction complex and the expression of R-spondin 2 potentiating Wnt signaling. When we measured Wnt/β-catenin signaling activity using a β-catenin/TCF reporter system we observed that cell lines do show a different Wnt-pathway activation upon exposure to Wnt ligand with high activity in ST88-14 and 90-8TL and low activity in sNF96.2. This difference may be caused by the variable expression levels of Wnt pathway genes in different cell lines as reported by Luscan *et al*.^39^. Transient inhibition of both miR-135b and miR-889 using antisense inhibitors reduced the capacity of ST88-14 to induce Wnt signaling upon stimulation. In agreement with this observation is the fact that miR-135b and miR-889 inhibitors impair invasion of ST88-14 cells as Wnt signaling has been shown to be involved in invasion in many cancer cells^50^. No clear effects were seen on proliferation and migration of this cell line upon miR-135b and miR-889 inhibition. It cannot be excluded that the inhibition of Wnt signaling by interfering with miR-135b and miR-889 levels is not potent enough to affect these processes.

An intriguing question is what causes the aberrant expression of miRNAs as seen in MPNST. It was recently reported that in about 60% of NF1-derived MPNST the PRC2 complex is inactivated^44–46^. The PRC2 complex is a well-known epigenetic modulator of gene expression^51^ by establishing di-and trimethylation of histon H3 lysine 27 (HeK27me2 and HeK27me3) both critical epigenetic silencing marks. Inactivation of PRC2 leads to aberrant gene expression due to the loss of these silencing marks. A list of genes differentially expressed in MPNSTs with loss of PRC2 and those with wild-type PRC2 is presented by Lee *et al*.^45^. Interestingly the expression of *LEMD1*, the gene that harbors miR-135b in one of its introns, is also induced upon PRC2 inactivation. This may explain the clearly increased levels of miR-135b observed in at least some of the MPNST samples (Fig. 1, Supplementary Tables 4 and 5) as it is co-expressed with its host gene *LEMD1*. Of note, the increased miR-135b levels may enhance Wnt signaling activity in the MPNST cells. Likewise, PRC2 inactivation may affect the miRNA cluster on chromosome 14 that contains four of the miRNAs, including miR-889, that were found upregulated in MPNST. However, PRC2 inactivation does not readily explain the downregulation of miRNAs observed in MPNST. Interesting in this respect are findings of de Raedt *et al*. who demonstrated that inactivation of PRC2 boosts the Ras signaling pathway which is already activated by the NF1 loss in these tumors^44^. Ras activation has been reported to downregulate the expression of miR143/145 cluster^31^ thereby explaining their relatively low levels in MPNST.

Our findings indicate that miRNAs operate in a cell line specific manner as different NF1-associated MPNST cell lines respond to miRNA modulation with different intensities or in a different fashion. It could be that the cell lines that comprise our MPNST cell line panel differ at a molecular level, perhaps reflecting different chromosomal copy number alterations as commonly observed in MPNST samples^52^. This may cause the cell lines to respond differently to miRNA regulation. This is a highly relevant issue which is often overlooked, as usually only a limited number of cell lines is used in *in vitro* experiments to functionally characterize miRNAs. We have studied the cellular effects by modulating the levels of individual miRNAs transiently. It could very well be that miRNAs display additive, or even synergistic effects and give rise to more pronounced cellular phenotypes when their levels are modulated simultaneously.

## Conclusions

From our study we conclude that at least some miRNAs play essential regulatory roles in MPNST facilitating tumor progression. These, and other miRNAs that are aberrantly expressed in MPNST, may be exploited as biomarker, with miRNA presence and/or levels being measured in suspect plexiform neurofibroma biopsies or in the circulation where they may signal the presence of MPNST. These avenues should be explored and can be particularly valuable in the context of neurofibromatosis type 1, with patients having a 10–13% life time risk of developing MPNST. Finally, as miRNAs are powerful regulatory biomolecules their therapeutic potential should be investigated in the context of MPNST in addition to the exact biochemical pathways and genes they regulate. These investigations may identify novel drug targets and lead to more effective therapeutic strategies.

## Supplementary information

Supplementary information.

## Data Availability

All data generated or analysed during this study are included in this published article and in its Supplementary Information Files. The miRNA expression datasets generated and analysed during the current study have been deposited to the Gene Expression Omnibus (GEO) data repository under accession number GSE140987.
