# Supplementary material for: Deregulated microRNAs in neurofibromatosis type 1 derived malignant peripheral nerve sheath tumors
Source: Sci Rep. 2020 Feb 19;10:2927. doi: 10.1038/s41598-020-59789-4 (PMC7031337; doi:10.1038/s41598-020-59789-4)
Supplement: Supplementary file 1 — Supplementary information. [file 41598_2020_59789_MOESM1_ESM.pdf]

Supplementary Data

Deregulated microRNAs in neurofibromatosis type 1 derived malignant peripheral nerve sheath tumors

Azadeh Amirnasr[1], Robert M. Verdijk[2] , Patricia F. van Kuijk[1], Pinar Kartal[1], Anne L.M. Vriends[1], Pim J. French[3], Martin E. van Royen[4], Walter Taal[5], Stefan Sleijfer[1], Erik A.C. Wiemer[1*]

1. Dept. of Medical Oncology, Erasmus MC Cancer Institute, Erasmus University Medical Center, Rotterdam, The Netherlands
2. Dept. of Pathology, Erasmus University Medical Center, Rotterdam, The Netherlands
3. Department of Neurology, Cancer Treatment Screening Facility (CTSF), Erasmus University Medical Center, Rotterdam, The Netherlands
4. Department of Pathology, Cancer Treatment Screening Facility (CTSF), Erasmus Optical Imaging Centre (OIC), Erasmus University Medical Center, Rotterdam, The Netherlands.
5. Dept. of Neuro-oncology/Neurology, Erasmus University Medical Center, Rotterdam, The Netherlands

Correspondence:

Erik A.C. Wiemer Ph.D.

Department of Medical Oncology / Erasmus MC Cancer Institute

Erasmus University Medical Center / Room Be422

Wytemaweg 80

3015 CN Rotterdam, The Netherlands

Tel: +31-10-704 4357

Fax: +31-10-704 1053

Email: e.wiemer@erasmusmc.nl

Supplementary Table and Figure Legends

Supplementary Table 1 - Patient and tumor characteristics related to the FFPE plexiform neurofibroma and MPNST samples pairs.

Supplementary Table 2 - Patient and tumor characteristics related to the FFPE sporadic MPNST samples.

Supplementary Table 3 - Patient and tumor characteristics related to the fresh frozen plexiform neurofibroma, atypical neurofibroma and MPNST samples.

Supplementary Table 4 – Differentially expressed miRNAs between plexiform neurofibroma and MPNST. Listed are all 90 miRNAs ($p<0.025$; FDR<10%) that are differentially expressed between plexiform neurofibroma (NF) and MPNST and that were used in the supervised hierarchical clustering (Figure 1). P-values of T-test and false discovery rate p-values (FDR) are listed, as well as the fold-change in miRNA expression and the percentage of samples in which the expression of a specific miRNA was detected.

Supplementary Table 5 – Fold-difference up or down-regulation of selected microRNAs in plexiform neurofibroma-MPNST sample pairs. Listed are the fold-differences of the downregulation of miR-145-5p, let-7b-5p, miR-143-3p, miR-139-5p and the upregulation of miR-135b-5p and miR-889-3p in MPNST vs plexiform neurofibroma (NF) observed in the different sample pairs analyzed in Figure 1. The fold-differences were calculated based on normalised expression values as deposited to the Gene Expression Omnibus (GSE 140987)

Supplementary Table 6 – Differentially expressed miRNA between NF1-associated MPNST and sporadic MPNST. Listed are all 45 miRNAs ($p<0.011$; FDR<10%) that are differentially expressed between NF1-associated MPNST (nMPNST) and sporadic MPNST (sMPNST) and that were used in the supervised hierarchical clustering (Figure 3). P-values of T-test and false discovery rate p-values (FDR) are listed, as well as the fold-change in miRNA expression and the percentage of samples in which the expression of a specific miRNA was detected.

Supplementary Table 7 - Differentially expressed microRNAs between neurofibroma and MPNST found in multiple studies.

Supplementary Fig. 1 – Cell line authentication by short tandem repeat analysis.
Short tandem repeat (STR) profiles were generated from the neurofibroma/MPNST cell line panel consisting of Hs53.T (cutaneous neurofibroma), sNF96.2 (NF1-associated MPNST), ST88-14 (NF1-associated MPNST), 90-8TL (NF1 associated MPNST) and STS26T (sporadic MPNST). Note that two batches of 90-8TL were analyzed obtained from different European laboratories. (B); dr. Eduard Serra, Institute of Predictive and Personalized Medicine of Cancer/IMPPC, Barcelona, Spain. (L); dr. Eric Legius, Department of Human Genetics, University Hospitals Leuven, Leuven, Belgium.

Supplementary Fig. 2 – Transfection efficiency control. Transfection conditions were optimized for each cell line using a fluorescently labelled miRNA mimic (miRIDIAN mimic transfection control Dy547; Dharmacon). The panel indicates the transfection efficiency observed in ST88-14, 90-8TL and sNF96.2 using the established transfection conditions. The left-hand phase-contrast panels highlight the individual cells, the right-hand panels indicate the

fluorescent signal detected in the same cells derived from the transfected fluorescently labelled mimic. The transfection effciency is estimated to be > 90% for all cell lines investigated.

Supplementary Fig. 3 – Unsupervised hierarchical clustering of paired plexiform neurofibroma and MPNST samples based on miRNA expression. Cluster tree showing the sample distribution from an unsupervised cluster analysis of paired (n=9) plexiform neurofibroma (NF) and MPNST samples based on the expression of all detectable miRNAs. The sample distribution indicates that most MPNST and plexiform neurofibromas (NF) cluster in separate branches of the cluster tree. Colored boxes indicate the neurofibroma/MPNST sample pairs with each individual pair derived from the same neurofibromatosis type 1 patient.

Supplementary Fig. 4 - Unsupervised hierarchical clustering of NF1-associated MPNST and sporadic MPNST samples based on miRNA expression. Cluster tree showing the sample distribution from an unsupervised cluster analysis of sporadic MPNST (S) (n=10) and NF1-associated MPNST (MPNST) samples based on the expression of all detectable miRNAs. The sample distribution indicates that sporadic MPNST and NF-1-associated MPNST predominantly cluster in distinct branches of the cluster tree.

Supplementary Fig. 5 – Cellular proliferation is not significantly affected in MPNST cell lines transfected with selected miRNA mimics and inhibitors. The NF1-associated MPNST cell lines sNF96.2, ST88-14 and 90-8TL were transfected with scrambled (LNA control), miR-135b and miR-889 inhibitors or with a scrambled (mneg), miR-143, miR-145, let-7b and miR-29c mimics. The bar graph depicts the percentage of viable cells at 72h post-transfection measured by SRB assay compared to the controls (LNA and mneg) which are arbitrarily set at

100%. Shown are mean values, error bars indicate SD (n=4). A Mann Whitney U test was used to determine statistical significance, p value <0.05 (*).

Supplementary Fig. 6 – Migration, invasion and Wnt/ß-catenin signaling activity are not affected in the MPNST cell line 90-8TL transfected with selected miRNA mimics and inhibitors. The NF1-associated MPNST cell line 90-8TL was transfected with scrambled (LNA control), miR-135b and miR-889 inhibitors or with a scrambled (mneg), miR-143, miR-145, let-7b and miR-29c mimics. (A) Scratch assay after which cell migration is monitored every two hours for 26 h using a live-cell imaging system (IncuCyte; Essen Bioscience Ltd.). Depicted are representatives graphs displaying average values, error bars indicate SD. (B) Invasion assay, invading cells are monitored every two hours for 67 h using a live-cell imaging system (IncuCyte). Y-axis indicates the “Total phase object area normalized to the initial top value” as a measure for the invading cell population. Depicted are representatives graphs displaying average values, error bars indicate SD. (C) Wnt/ß-catenin signaling activity upon induction by Wnt ligand was determined using a β-catenin/TCF reporter assay in 90-8TL cells transfected with a scrambled control inhibitor, a miR-135b or miR-889 inhibitor. Depicted are average values ± SD (n=9).

Supplementary Fig. 7 – Average cell speed as a measure for migratory capability. Average cell speeds were determined in the NF1-associated MPNST cell lines sNF96.2, ST88-14 and 90-8TL after transfection with a scrambled control mimic (mneg control) and mimics for let-7b-5p, miR-145-5p and miR-29c-3p. Twenty-four hours after transfection the cells were monitored at 2 h intervals using an advanced live cell imaging platform (Opera Phenix™, PerkinElmer) for 40 h. Depicted are Box-Whisker plots with the boxes showing 1st to 3rd quartile with the median marked as horizontal line. Each red dot indicates the calculated average

speed in μm/s at each 2 h interval of all cells present in a single well. Only overexpression of let-7b and miR-29c significantly reduced average cell speeds in sNF96.2 and ST88-14, respectively. A T-test was used to determine statistical significance, p value <0.01 (**).

Paired FFPE tumor samples (n = 9 pairs)	
**Gender**	
Male	6 (66.7 %)
Female	3 (33.3 %)
**Age at biopsy/resection NF (years)**	
Median (range)	28 (5 - 63)
**Age at biopsy/resection MPNST (years)**	
Median (range)	27 (14 - 70)
**Plexiform neurofibroma**	
Head and Neck	1 (11.1 %)
Extremities	3 (33.3 %)
Trunk	5 (55.6 %)
**MPNST (NF1-derived)**	
Head and Neck	1 (11.1 %)
Extremities	4 (44.4 %)
Trunk	4 (44.4 %)

MPNST; malignant peripheral nerve sheath tumor
NF; plexiform neurofibroma

Supp table 1

FFPE tumor samples (n = 10)	
**Gender**	
Male	5 (50 %)
Female	5 (50 %)
**Age at biopsy/resection MPNST (years)**	
Median (range)	37 (10 - 71)
**MPNST (sporadic)**	
Head and Neck	3 (30 %)
Extremities	2 (20 %)
Trunk	5 (50 %)

MPNST; malignant peripheral nerve sheath tumor

Supp table 2

Fresh Frozen tumor samples	
**Plexiform neurofibromas (n = 7)**	
**Gender**	
Male	4 (57.1 %)
Female	3 (42.9 %)
**Age at biopsy/resection (years)**	
**Median (range)**	29(10 - 63)
**Location**	
Head and Neck	1 (14.3 %)
Extremities	4 (57.1 %)
Trunk	2 (28.6 %)
**Atypical neurofibromas (n = 4)**	
**Gender**	
Male	2 (50 %)
Female	2 (50 %)
**Age at biopsy/resection (years)**	
**Median (range)**	25.5 (15 - 43)
**Location**	
Head and Neck	-
Extremities	4 (100 %)
Trunk	-
**MPNST (NF1-derived; n = 11)**	
**Gender**	
Male	5 (45.5 %)
Female	6 (54.5 %)
**Age at biopsy/resection (years)**	
**Median (range)**	36 (12 - 76)
**Location**	
Head and Neck	3 (27.3 %)
Extremities	3 (27.3 %)
Trunk	5 (45.4 %)

MPNST; malignant peripheral nerve sheath tumor
NF; plexiform neurofibroma

Supp table 3

	miRNA	Up in MPNST vs NF	Down in MPNST vs NF	% detection	Parametric P value	FDR
1	hsa-miR-145#		7.085353227	100%	2.43553E-06	0.000735575
2	hsa-miR-145		15.06757956	100%	3.99769E-06	0.000735575
3	hsa-miR-139-5p		21.69731072	100%	1.28601E-05	0.00157751
4	hsa-miR-889	3.406335838		94%	3.03802E-05	0.00279498
5	hsa-miR-126		4.726451114	100%	5.80149E-05	0.004139758
6	hsa-miR-150		37.12611354	83%	6.74961E-05	0.004139758
7	hsa-miR-363		3.978428028	94%	9.05171E-05	0.004758614
8	hsa-miR-28-3p		4.437191461	94%	0.000153331	0.006890113
9	hsa-miR-143		6.465421082	94%	0.000168508	0.006890113
10	hsa-miR-338-3p		39.70932342	83%	0.000216324	0.007960718
11	hsa-miR-541	20.29148001		72%	0.000247917	0.008293949
12	hsa-miR-95		10.56783787	100%	0.00035673	0.009407262
13	hsa-let-7b		7.587002431	100%	0.000364975	0.009407262
14	hsa-miR-30e-3p		4.734986933	100%	0.000372741	0.009407262
15	hsa-miR-135b	51.7127516		94%	0.000383448	0.009407262
16	hsa-miR-27a#		17.66501109	83%	0.000478538	0.009983324
17	hsa-miR-328		6.609667354	100%	0.0005228	0.009983324
18	hsa-miR-223#		7.575083428	100%	0.000528457	0.009983324
19	hsa-miR-24		3.799034698	100%	0.000533456	0.009983324
20	hsa-miR-223		5.658724891	100%	0.000548501	0.009983324
21	hsa-miR-185		3.595079758	100%	0.000569701	0.009983324
22	hsa-miR-125b		3.315971381	100%	0.000619511	0.010362727
23	hsa-miR-126#		4.181460367	100%	0.000684774	0.010956381
24	hsa-miR-26b#		5.662552199	100%	0.000964193	0.014315879
25	rno-miR-29c#		11.03628082	89%	0.000972546	0.014315879
26	hsa-miR-146a		18.80394044	100%	0.001055119	0.014933992
27	hsa-miR-140-3p		6.388086065	94%	0.001188825	0.016141999
28	hsa-miR-489		12.97692807	100%	0.001228196	0.016141999
29	hsa-miR-34b		5.706772015	89%	0.001581652	0.020070625
30	hsa-miR-886-5p		7.498248067	100%	0.001678769	0.020592897
31	hsa-miR-99a#		4.444834732	100%	0.001861343	0.021476176
32	hsa-miR-511		8.083615307	100%	0.001867494	0.021476176
33	hsa-miR-30a-3p		5.076990067	89%	0.002139978	0.023864
34	hsa-miR-574-3p		3.595169192	100%	0.002217328	0.023999311
35	hsa-miR-29c		8.617226868	100%	0.002315022	0.024340802
36	hsa-miR-885-5p		8.879616276	100%	0.002732209	0.027929248
37	hsa-miR-184		10.82249245	100%	0.00287859	0.028630302
38	hsa-miR-886-3p		6.704227524	100%	0.003055147	0.029586686
39	hsa-miR-191		3.443459664	100%	0.003419799	0.032268874
40	hsa-miR-27b		3.061650636	100%	0.004027826	0.037056
41	hsa-let-7d		4.005503366	100%	0.004135533	0.037118926
42	hsa-miR-18a	6.149777119		83%	0.004367727	0.038215212
43	hsa-miR-100		3.1650557	100%	0.004518766	0.038215212
44	hsa-miR-151-5P		4.286341774	100%	0.00456921	0.038215212
45	hsa-miR-548c		5.200739304	89%	0.005318592	0.043494267
46	hsa-miR-628-5p		2.483228656	100%	0.00553844	0.044307521
47	hsa-miR-193a-5p		3.255621041	100%	0.005744203	0.044975884
48	hsa-miR-197		3.735489964	94%	0.006363588	0.048787509
49	hsa-miR-26a		5.362360273	89%	0.006656797	0.049077627
50	hsa-miR-34a		4.601277782	100%	0.006675247	0.049077627
51	hsa-miR-29a		9.12510384	72%	0.006801519	0.049077627
52	hsa-miR-331		2.813574179	100%	0.006935456	0.049081687
53	hsa-miR-532-3p		2.890506756	100%	0.00715722	0.049695413
54	hsa-miR-204		8.896565878	100%	0.007529147	0.051309739
55	hsa-miR-144#		4.008534108	78%	0.0079326	0.051663679
56	hsa-miR-26a-1#		4.273740238	100%	0.007980629	0.051663679
57	hsa-miR-340		4.379681628	94%	0.008002255	0.051663679
58	hsa-miR-433	2.394760055		100%	0.008400196	0.053297794
59	hsa-let-7e		3.776960732	94%	0.008588059	0.053566195
60	hsa-miR-34a#		3.804526148	100%	0.009218203	0.056538314
61	hsa-miR-27a		3.314832628	94%	0.009699776	0.058516684
62	hsa-let-7a		3.269662019	94%	0.010314464	0.059832
63	hsa-let-7c		4.480415537	94%	0.010323084	0.059832
64	hsa-miR-339-5p		3.427683059	61%	0.010405565	0.059832
65	hsa-miR-29b-2#		11.63503844	78%	0.010836735	0.061073297
66	hsa-miR-1274B		3.243060265	100%	0.011145673	0.061073297
67	hsa-miR-29b		8.409008691	94%	0.011167451	0.061073297
68	hsa-miR-30c		3.891187071	94%	0.011285283	0.061073297
69	hsa-miR-151-3p		2.762567832	100%	0.011753067	0.062683026
70	hsa-miR-642		3.237932202	100%	0.012804183	0.065499095
71	hsa-miR-1256		3.821234454	100%	0.012813869	0.065499095
72	hsa-miR-186		2.754394108	100%	0.01281504	0.065499095
73	hsa-miR-16		2.828497051	94%	0.013850471	0.069198091
74	hsa-miR-200c		14.70615801	94%	0.013914834	0.069198091
75	hsa-miR-520D-3P		3.001842776	94%	0.014142404	0.06939206
76	hsa-miR-519d	6.785737397		56%	0.014465832	0.07004508
77	hsa-miR-505#		2.057819344	100%	0.015536907	0.074105079
78	hsa-miR-330		3.344768821	89%	0.015707055	0.074105079
79	hsa-miR-720		3.616158437	100%	0.01715492	0.079911526
80	hsa-miR-99a		3.138284622	94%	0.017579167	0.080864169
81	hsa-miR-222		2.812197607	83%	0.018152005	0.08246837
82	hsa-miR-193b		2.719451654	100%	0.018529078	0.083118207
83	hsa-miR-423-5p		4.124111259	89%	0.01874677	0.083118207
84	hsa-miR-340#		3.31515553	100%	0.019193526	0.084085923
85	hsa-miR-493	1.77437126		94%	0.021245382	0.091980007
86	hsa-miR-210	1.8257531		94%	0.023362142	0.099614269
87	hsa-miR-133a		5.178886139	94%	0.023914448	0.099614269
88	mmu-miR-140		2.462767136	100%	0.024065364	0.099614269
89	hsa-miR-26a-2#		3.771382909	89%	0.024141724	0.099614269
90	hsa-miR-30a-5p		3.464006666	89%	0.024362185	0.099614269

Supp table 4

Fold-difference up or down-regulation of selected microRNAs in NF-MPNST sample pairs

	Down in MPNST vs NF				Up in MPNST vs NF	
	**miR-145-5p**	**let-7b-5p**	**miR-143-3p**	**miR-139-5p**	**miR-135b-5p**	**miR-889-3p**
**Pair 1**	22.63	15.67	14.51	11.61	-	-
**Pair 2**	14.65	16.47	4.18	41.36	362.80	4.52
**Pair 3**	17.38	28.46	-	64.86	4.13	2.62
**Pair 4**	19.98	5.17	7.05	37.94	9.16	4.78
**Pair 5**	16.03	9.35	7.15	20.68	932.41	4.50
**Pair 6**	2.23	1.45	1.59	2.37	12.83	3.69
**Pair 7**	22.40	18.45	7.37	37.96	77.20	1.93
**Pair 8**	17.83	2.24	3.88	20.00	376.40	4.97
**Pair 9**	24.35	3.91	11.45	25.40	55.36	7.40

Supp table 5

	miRNA	Up in sMPNST vs nMPSNT	Down in sMPNST vs nMPSNT	% detection	Parametric P value	FDR
1	hsa-miR-19b-1#	373.9411		95%	0.00000	4.55E-06
2	hsa-miR-342-3p	30.2649		100%	0.00000	8.77E-06
3	hsa-miR-340		32.0428	100%	0.00000	9.4E-06
4	hsa-miR-518b		762.0100	68%	0.00001	0.000838
5	mmu-miR-374-5p	111.0402		89%	0.00001	0.001075
6	hsa-miR-628-5p		136.3182	100%	0.00002	0.001114
7	hsa-miR-186		3.7736	100%	0.00004	0.002347
8	hsa-miR-518f		22595.2383	89%	0.00005	0.002745
9	hsa-miR-218		6.8093	100%	0.00007	0.003367
10	hsa-miR-604		59.3902	84%	0.00010	0.004068
11	hsa-miR-551b		198.0057	100%	0.00022	0.007684
12	hsa-miR-375		461.8530	84%	0.00024	0.007684
13	hsa-miR-378		437.5465	95%	0.00024	0.007684
14	hsa-miR-27a#	23.9460		100%	0.00028	0.007778
15	hsa-miR-519b-3p		39.5141	79%	0.00029	0.007778
16	hsa-miR-338-5P	31.0899		58%	0.00031	0.007778
17	hsa-miR-888		243.8921	63%	0.00032	0.007778
18	hsa-miR-26b#		8.6038	95%	0.00034	0.007779
19	hsa-miR-222#	13.4332		95%	0.00040	0.008679
20	hsa-miR-203		19.7964	100%	0.00047	0.009689
21	hsa-miR-26a-2#	9.8854		95%	0.00066	0.013151
22	hsa-miR-1285	252.2160		89%	0.00086	0.016175
23	hsa-miR-629	10.7002		89%	0.00096	0.017391
24	hsa-miR-590-5p		12.8421	95%	0.00115	0.019931
25	hsa-miR-500	251.4972		79%	0.00157	0.026127
26	hsa-miR-34b	16.7749		89%	0.00166	0.026202
27	rno-miR-7#		8.4749	79%	0.00170	0.026202
28	hsa-miR-487b		6.6345	100%	0.00227	0.0338
29	hsa-miR-365	6.2608		100%	0.00254	0.036334
30	hsa-miR-654-3p	38.7682		58%	0.00262	0.036334
31	hsa-miR-324-3p		3.3429	100%	0.00278	0.037371
32	hsa-miR-625	9.9808		95%	0.00375	0.048694
33	hsa-miR-370		8.3451	100%	0.00392	0.048759
34	hsa-miR-432		14.4270	95%	0.00399	0.048759
35	hsa-miR-539		10.3823	95%	0.00487	0.05761
36	hsa-miR-520D-3P		70.4517	100%	0.00499	0.05761
37	hsa-miR-345	4.6907		100%	0.00555	0.062437
38	hsa-miR-199a-3p		3.6918	100%	0.00616	0.06748
39	hsa-miR-454		3.3369	95%	0.00671	0.071619
40	mmu-miR-379		3.2205	89%	0.00729	0.075866
41	mmu-miR-496		11.7195	89%	0.00815	0.082717
42	hsa-miR-376a		8.4387	100%	0.00867	0.085857
43	hsa-miR-1243	19.0775		42%	0.00919	0.088891
44	hsa-miR-410		8.6207	100%	0.01010	0.095497
45	hsa-miR-548c-5p	3.4871		95%	0.01069	0.0988

Supp table 6

microRNA	Ref. 1	Ref. 2	Ref. 3	Ref. 4	Ref. 5
Upregulated in MPNST					
**miR-10b**		✓		✓	
**miR-18a**		✓			✓
**miR-135b**		✓			✓
**miR-210**	✓	✓	✓		✓
Downregulated in MPNST					
**Let-7a**			✓	✓	✓
**Let-7b**	✓	✓		✓	✓
**miR-16**	✓				✓
**miR-26a**	✓				✓
**miR-27a**	✓				✓
**miR-29a**	✓		✓		✓
**miR-29b**	✓		✓		✓
**miR-29c**	✓		✓		✓
**miR-29c***			✓		✓
**miR-30a-5p**	✓				✓
**miR-30c**			✓		✓
**miR-34a**	✓				✓
**miR-95**		✓			✓
**miR-99a**	✓				✓
**miR-100**	✓				✓
**miR-125b**	✓				✓
**miR-133a**	✓				✓
**miR-139-5p**		✓	✓		✓
**miR-143**	✓				✓
**miR-145**	✓				✓
**miR-146a**	✓	✓	✓		✓
**miR-150**		✓	✓		✓
**miR-151-5p**			✓		✓
**miR-186**		✓			✓
**miR-195**	✓	✓	✓		
**miR-200c**		✓			✓
**miR-222**	✓				✓
**miR-223**	✓				✓
**miR-338-3p**	✓	✓			✓
**miR-885-5p**		✓			✓

Ref. 1 – Subramanian, S. et al. (2010) J. Pathol. 220:58-70; Ref. 2 – Masliah-Planchon, J. et al. (2013) BMC Genomics 14:473; Ref. 3 – Presneau, N. et al. (2013) Br. J. Cancer 108:964-972; Ref. 4 – Chai, G. et al. (2010) Cancer Sci. 101:1997-2004; Amirnasr, A. et al. (2017) current study

Supp table 7

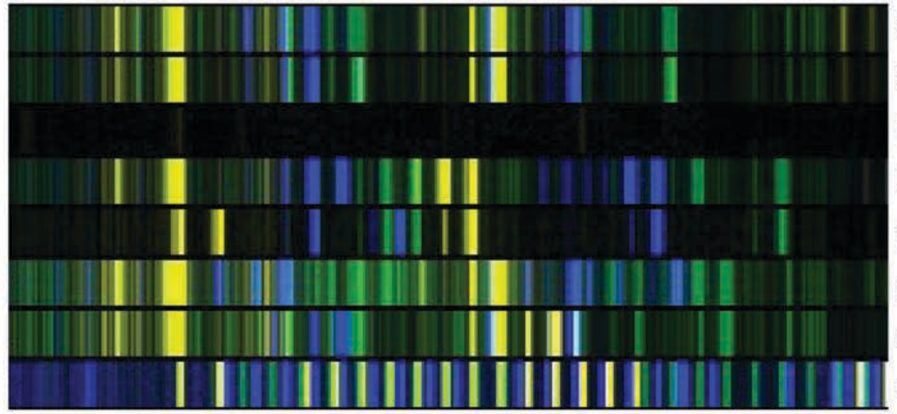

cell line	D3S1358	TH01	D21S11	D18S51	Penta_E	D5S818	D13S317	D7S820	D16S539	CSF1PO	Penta_D	Amelogenin	vWA	D8S1179	TPOX	FGA
90-8TL (B)	15,21	6	30	14,18	5,10	11	8	11	9	12	14	X	17	10,12	11	25
90-8TL (L)	15,21	6	30	14,18	5,10	11	8	11	9	12	14	X	17	10,12	11	25
Hs53.T	15,16	8,9	27,29	18,19	8,9	12,13	9,12	12	10,12	7,10	9,11	X	15,16	11,12	7,9	23,24
ST88-14	15,18	9	29,32.2	12	16	12,13	12	8	13	9,12	9,13	X,Y	16	14	11,12	21
STS26T	14	6,9.3	30,31	17,18	12,13	11,12	9,10	8.11	12,13	10,13	8,12	X	17	13,14	8	22,23
SNF96.2	16,21	6	30	16	15	11	10	10.11	11	12	14	X	17,19,20	13	11	22

Supp Fig.1

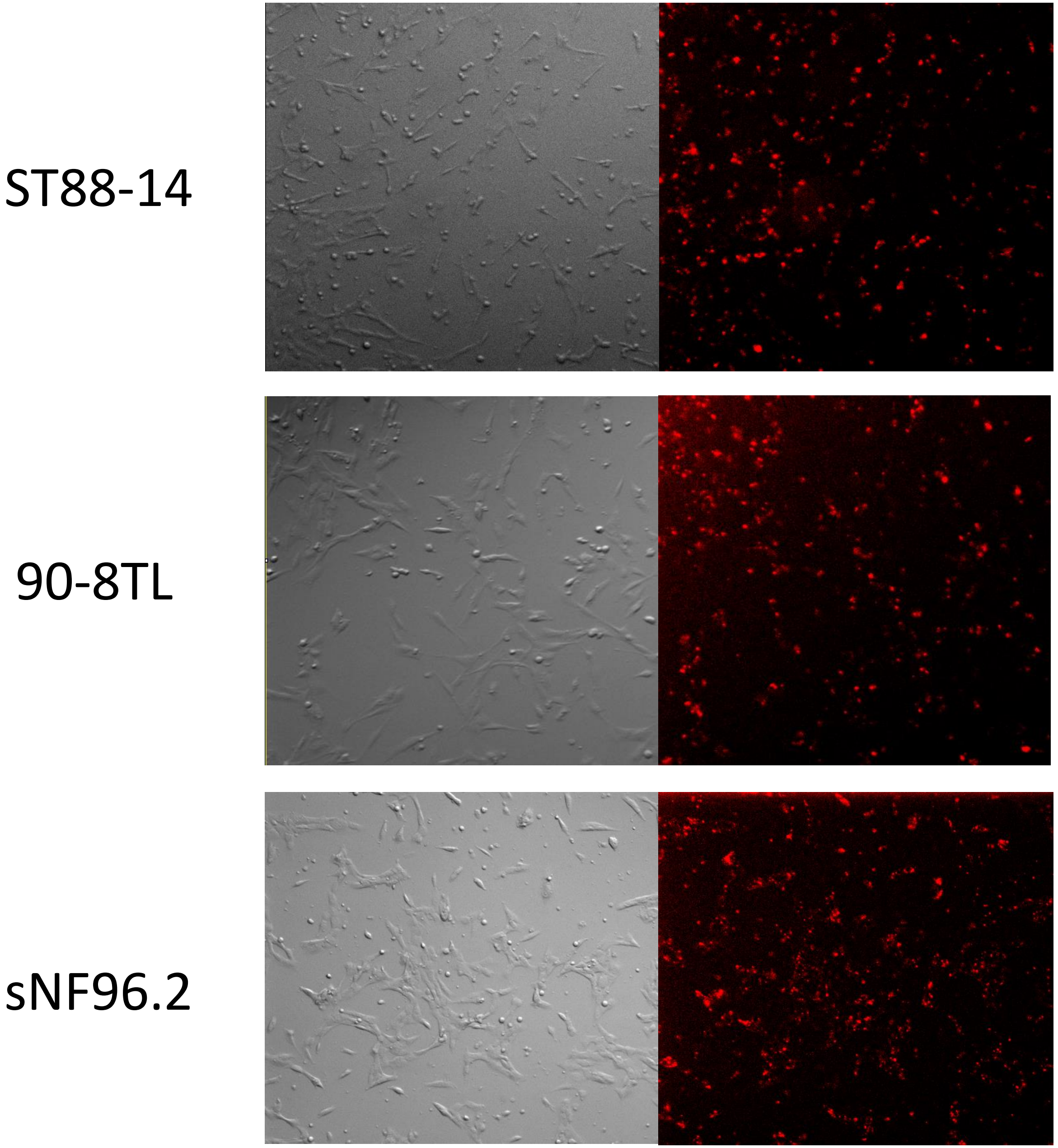

Supp. Fig. 2

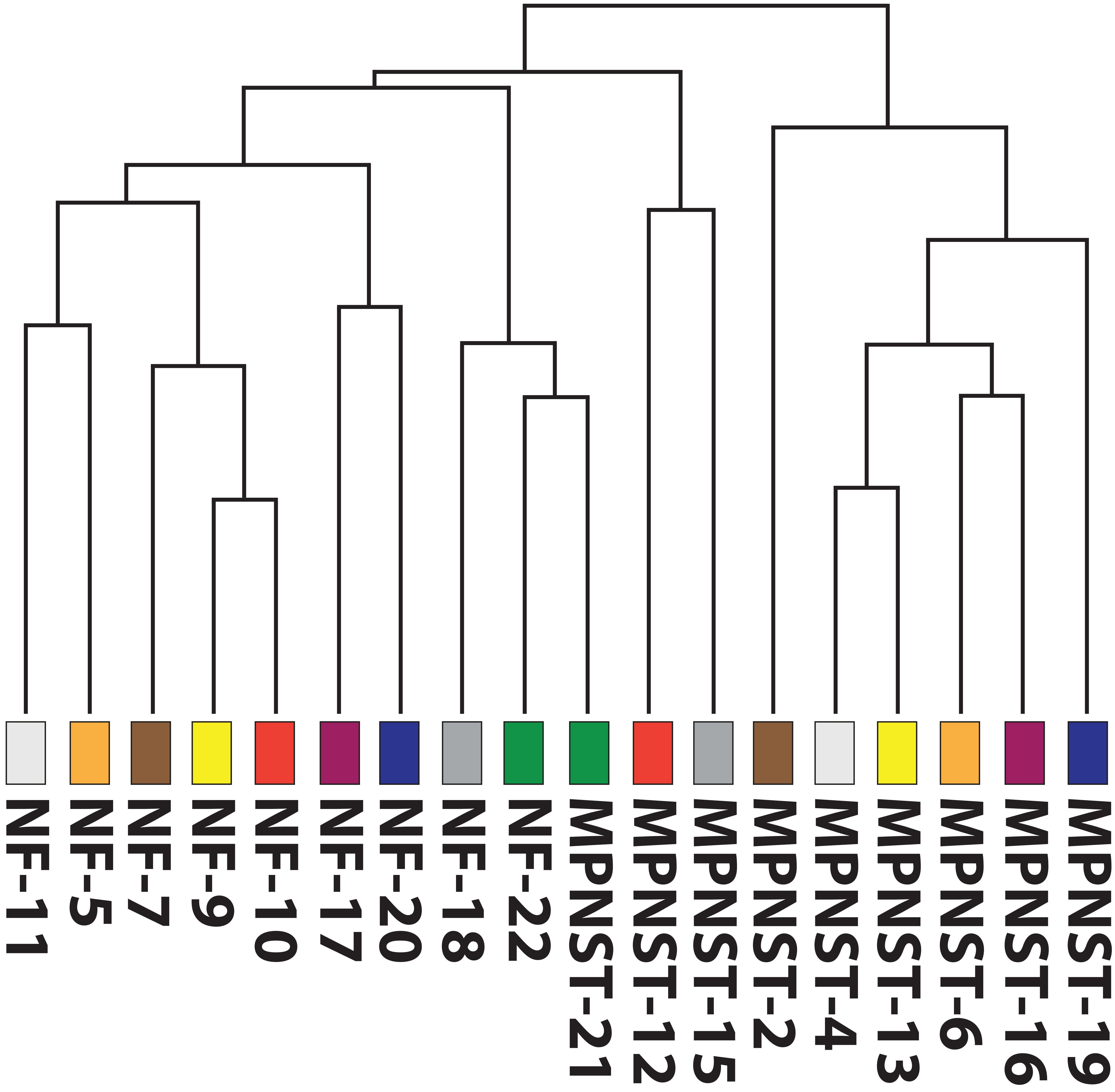
NF-11
NF-5
NF-7
NF-9
NF-10
NF-17
NF-20
NF-18
NF-22
MPNST-21
MPNST-12
MPNST-15
MPNST-2
MPNST-4
MPNST-13
MPNST-6
MPNST-16
MPNST-19

Supp Fig.3

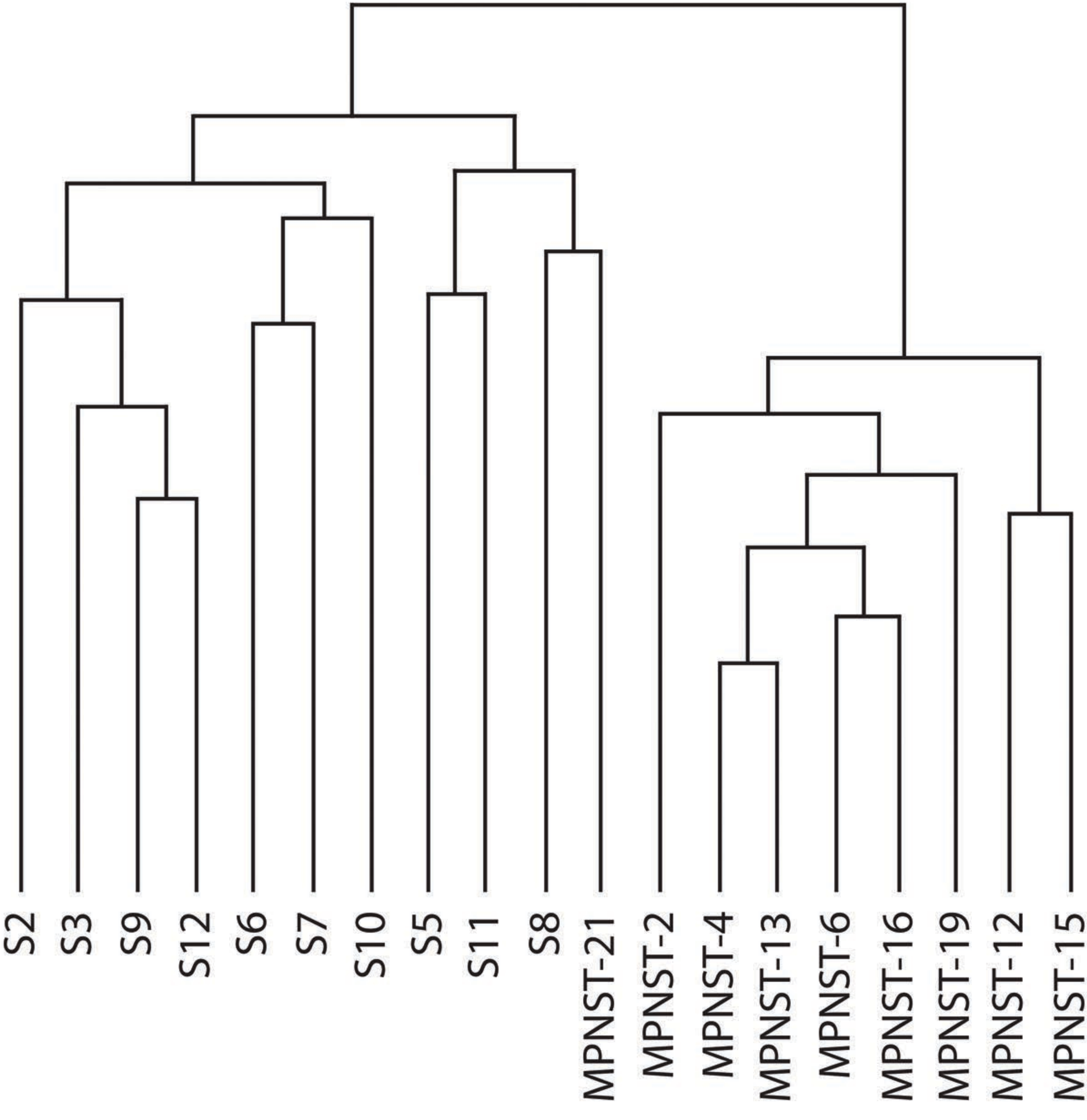
S2
S3
S9
S12
S6
S7
S10
S5
S11
S8
MPNST-21
MPNST-2
MPNST-4
MPNST-13
MPNST-6
MPNST-16
MPNST-19
MPNST-12
MPNST-15

Supp Fig.4

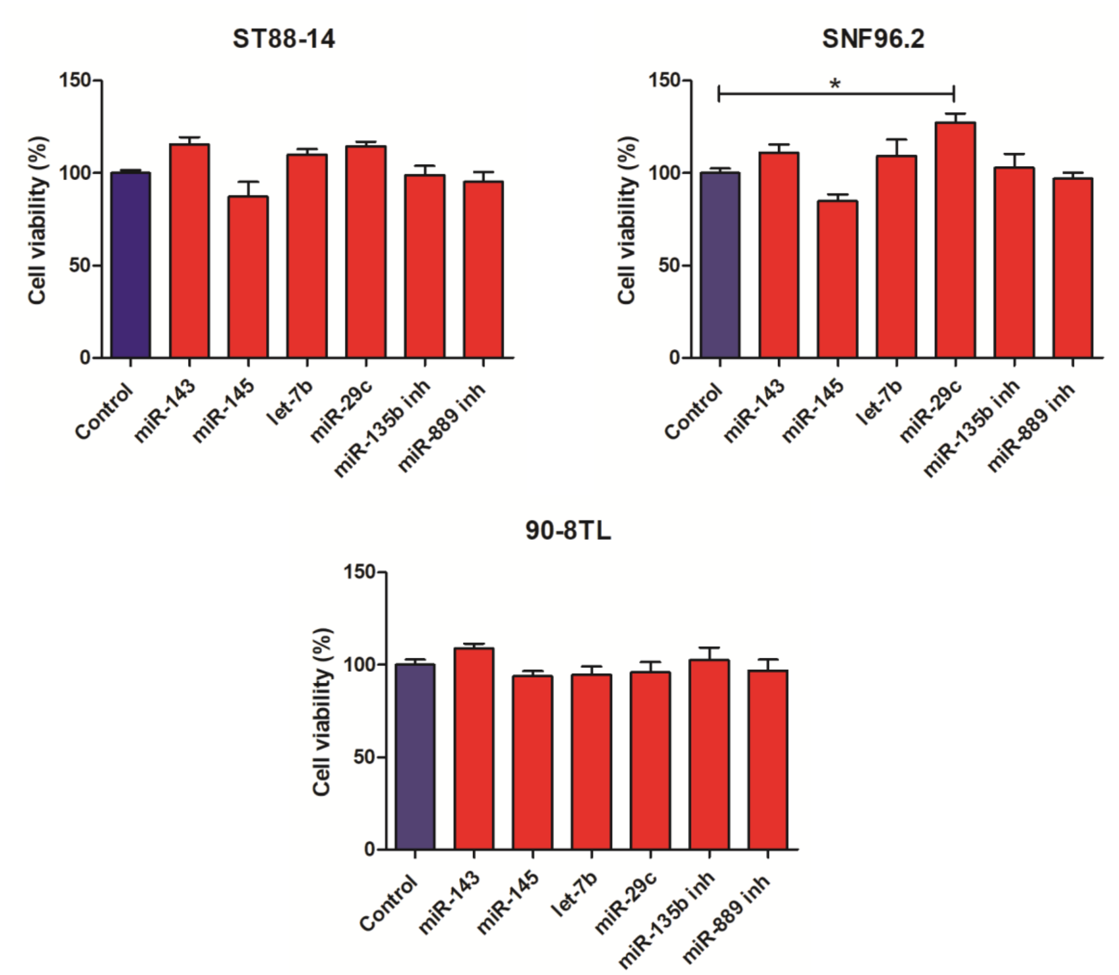
ST88-14
SNF96.2
90-8TL
Cell viability (%)
150
100
50
0
*
Control
miR-143
miR-145
let-7b
miR-29c
miR-135b inh
miR-889 inh

Supp Fig. 5

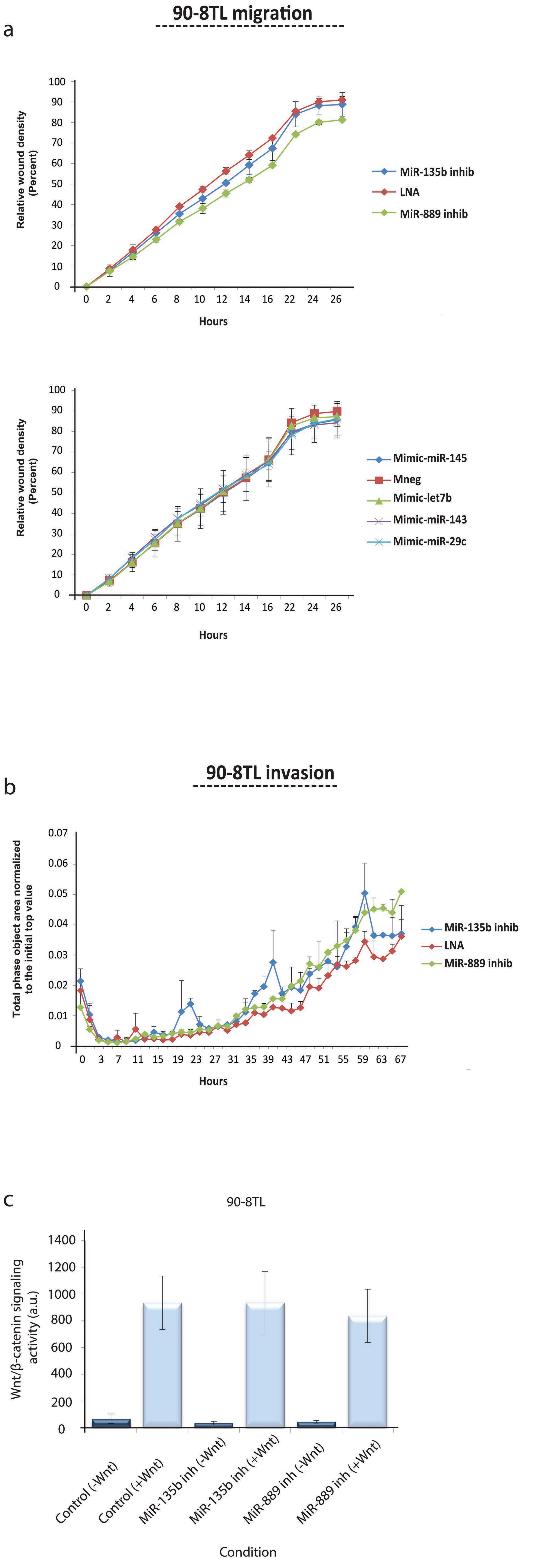
a
90-8TL migration
Relative wound density (Percent)
100
90
80
70
60
50
40
30
20
10
0
0 2 4 6 8 10 12 14 16 22 24 26
Hours
MiR-135b inhib
LNA
MiR-889 inhib
Mimic-miR-145
Mneg
Mimic-let7b
Mimic-miR-143
Mimic-miR-29c
b
90-8TL invasion
Total phase object area normalized to the initial top value
0.07
0.06
0.05
0.04
0.03
0.02
0.01
0
0 3 7 11 15 19 23 27 31 35 39 43 47 51 55 59 63 67
Hours
c
90-8TL
Wnt/β-catenin signaling activity (a.u.)
1400
1200
1000
800
600
400
200
0
Control (-Wnt)
Control (+Wnt)
MiR-135b inh (-Wnt)
MiR-135b inh (+Wnt)
MiR-889 inh (-Wnt)
MiR-889 inh (+Wnt)
Condition

Supp Fig.6

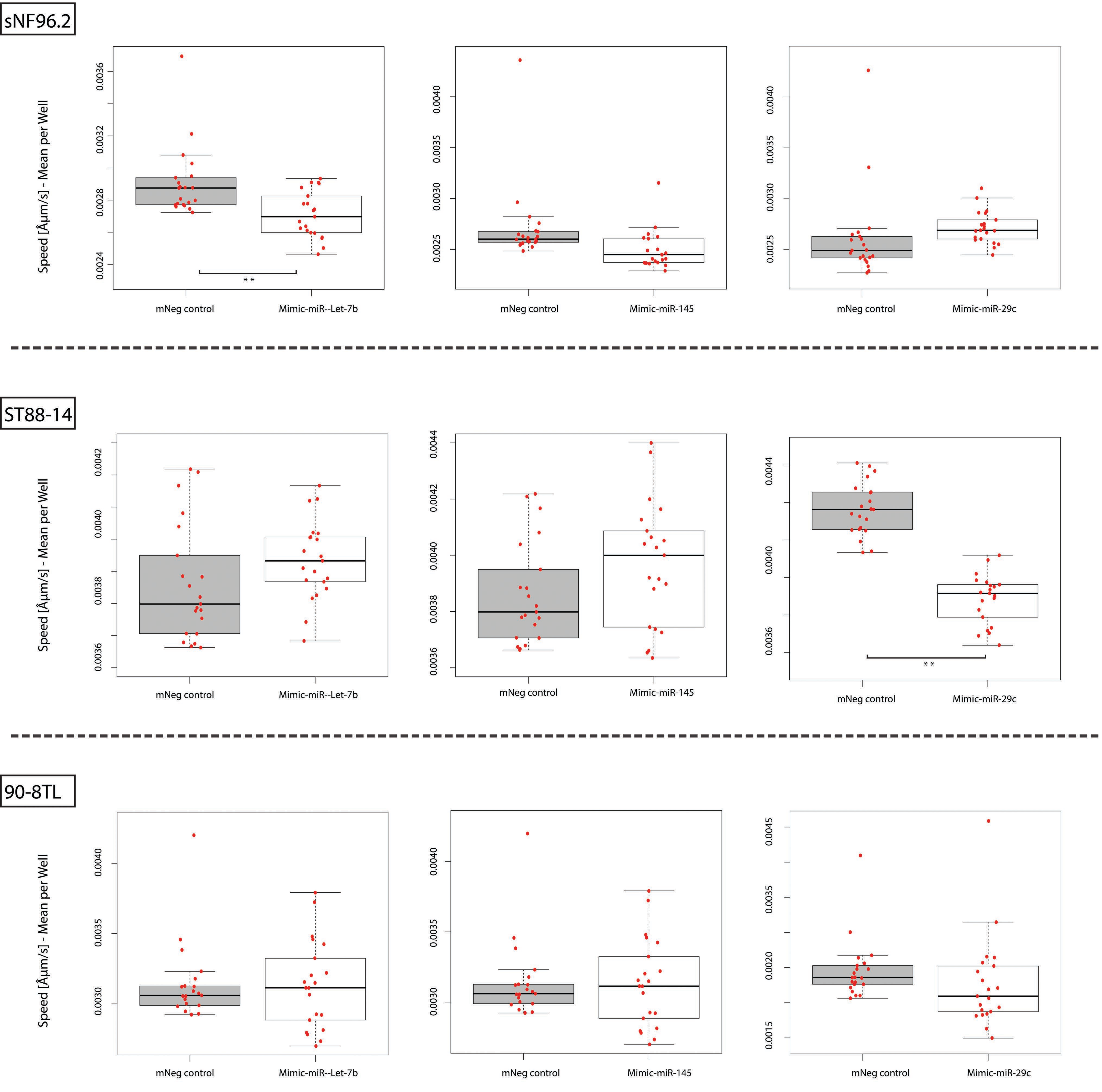
sNF96.2
ST88-14
90-8TL
Speed [Âµm/s] - Mean per Well
mNeg control
Mimic-miR--Let-7b
Mimic-miR-145
Mimic-miR-29c
**
0.0024
0.0028
0.0032
0.0036
0.0025
0.0030
0.0035
0.0040
0.0036
0.0038
0.0040
0.0042
0.0044
0.0015
0.0020
0.0045

Supp Fig.7
